# “An interpretative phenomenological analysis of male body image through the lived experiences of men in India”

**DOI:** 10.1186/s40359-025-02963-y

**Published:** 2025-07-01

**Authors:** Gerome Karthic Kumar Louis, Gandhapodi K. Chithra

**Affiliations:** Division of English - School of Social Sciences and Languages, Vellore Institute of Technology, Chennai, 600127 India

**Keywords:** Lived experiences, Cisgender men, Male body image, IPA, Stereotypes

## Abstract

**Purpose:**

This study aims to explore and analyze the lived experiences of cisgender men in India concerning male body image, employing an Interpretative Phenomenological Analysis (IPA) approach. It seeks to understand the psychological and sociocultural influences shaping their perceptions of body image, addressing the gap in the literature, which has focused primarily on Western contexts.

**Methodology:**

A qualitative research design involving in-depth interviews with a purposively selected sample of cisgender men from diverse backgrounds in India was utilized. The data were analyzed via IPA to identify group and personal experiential statements related to body image, self-perception, and societal expectations.

**Results:**

The findings revealed significant group experiential statements, including the impact of societal expectations, media representations, and peer influences on the participants' body image. The study highlighted the tension between traditional masculine ideals and modern body expectations, leading to varying degrees of body dissatisfaction and self-esteem issues among the participants.

**Conclusion:**

This research underscores the importance of contextualizing body image studies within specific cultural frameworks. This suggests the need for more inclusive and sociologically sensitive approaches in addressing stereotypes such as body image issues, particularly in non-Western contexts. The study contributes to the broader discourse on male body image by providing insights into the unique experiences of Indian men, with implications for future research and interventions.

**Supplementary Information:**

The online version contains supplementary material available at 10.1186/s40359-025-02963-y.

## Introduction

Body image refers to an individual's perception, thoughts, and feelings about their physical appearance. It encompasses not only the visual assessment of one’s body but also the emotional attitudes and thoughts associated with these perceptions. This concept is deeply tied to societal constructions of masculinity, which often emphasize ideals such as strength, muscularity, and self-control. These ideals exert pressure on men to conform to rigid physical standards, shaping their self-perceptions and behaviors. Masculinity, as a cultural construct, demands a particular embodiment that aligns with societal notions of male success, dominance, and desirability. In this context, body image becomes a critical site where the expectations of masculinity are negotiated and contested.

Theoretical frameworks such as the Tripartite Influence Model provide valuable insights into how body image concerns develop. According to this model, sociocultural factors—including media, peers, and family—play a pivotal role in shaping an individual’s body image [1]. Media representations, in particular, have a profound influence on men’s perceptions of their bodies. Advertisements, movies, and social media platforms frequently portray an idealized male physique characterized by hyper-muscularity, leanness, and athleticism. Studies such as those by Labre [2] and Galioto [3] have demonstrated that prolonged exposure to such media imagery can foster body dissatisfaction and drive behaviors aimed at achieving these often-unattainable standards.

Cultural norms further complicate this issue, as ideals of masculinity and attractiveness vary significantly across different societies. For instance, research by El Ansari et al. [4] highlights how cultural values and societal expectations shape body satisfaction differently across populations. While Western ideals often prioritize muscularity, other cultures may emphasize different attributes, such as agility or endurance. However, the influence of media has become increasingly globalized, propagating Westernized standards of male beauty to non-Western contexts. Despite a growing body of literature on male body image globally, there remains a notable lack of research focused on Indian men. This study addresses this gap by examining the intersections of culture, masculinity, and body image within the Indian sociocultural context.

In addition to sociocultural influences, psychological factors play a crucial role in shaping male body image. Perfectionism is particularly significant in this regard, as it involves striving for unattainable physical standards. Men who internalize such ideals often experience dissatisfaction, anxiety, and negative self-perceptions when they fail to meet these expectations [5]. The psychological toll of perfectionism has been linked to a range of issues, including depression, eating disorders, and substance use, as noted by Olivardia et al. [6] and Goldfield et al. [7]. These findings underscore the interplay between societal expectations and individual vulnerabilities, highlighting the multifaceted nature of body image concerns.

Subcultural influences further add to this complexity. Within certain subcultures, such as fitness communities or athletic groups, leanness and muscularity are highly valued, potentially reinforcing unrealistic body standards. Philips [8] explored how fitness subcultures can exacerbate body image concerns, while Baghurst [9] identified variations in muscle dysmorphia traits across different athletic disciplines. Such findings emphasize the need to consider both cultural and subcultural factors when studying male body image.

### Knowledge gap and research question

The literature highlights the influences of media, sociological factors, and gender-specific traits on male body image. However, it fails to address the unique ways in which these factors affect men compared with women. There is a gap in the understanding of the specific mechanisms that drive male body image concerns, as well as a need for more targeted psychological and behavioral interventions for men. The present study aims to explore the nuanced dimensions of male body image and offer a more comprehensive approach to understanding and addressing these issues. Additionally, most of the studies focus on Western populations, particularly college-aged men and athletes in the U.S., Europe, and Australia. For example, Olivardia et al. [6] and Kanayama et al. [10] examined American college men and steroid users, whereas Murray et al. [11] studied male muscle dysmorphia patients in Australia. Some studies [4, 12] explore cross-cultural contexts centered on non-Western populations with a limited scope, such as Egypt and Turkey. These demographics suggest a lack of research on male body image issues within the Indian context, highlighting the need to investigate how masculinity pressures and cultural factors uniquely shape body image concerns in India.

Research on Body Image Dissatisfaction (BID) in India has traditionally focused on women, while studies on men’s body image remain scarce. Several studies explore how women in India, influenced by sociocultural factors and media, experience body dissatisfaction. For example, Zimik’s sociological study [13] highlighted the pressures Indian women face due to idealized beauty standards, largely influenced by Western ideals. However, few studies investigate men’s body image, despite growing evidence that male body dissatisfaction is an issue in India.

Soohinda et al. [14] research on young Indian men stands out as one of the few exploring male BID. This study reveals that 34.44% of participants reported moderate to severe BID, influenced by sociocultural pressures from peers, media, and family. Interestingly, underweight men exhibited greater body dissatisfaction than obese men, suggesting a preference for muscularity over thinness. The study also linked BID with personality traits like neuroticism, but not with self-esteem or BMI.

This limited focus on male body image in India highlights the need for more research. Current literature has been centered on women’s body image issues, leaving a gap in understanding how sociocultural factors impact men. Soohinda et al. [14] underscores the rising influence of Western ideals in shaping male body perceptions in India, providing a foundation for future research. With male BID becoming an emerging issue, further exploration is crucial to understand its prevalence and psychological consequences, promoting better mental health interventions for men.

The research question formulated for the present study is “What specific experiences and challenges concerning body dissatisfaction, muscle dysmorphia, and the pursuit of muscularity ideals are identified by a sample of men living in India?”.

## Method

The researchers conducted a qualitative study using interpretative phenomenological analysis (IPA) as an analytic strategy to examine the real-life experiences of men regarding their"male body image."They adopted the IPA approach based on the methodology outlined by Smith and Osborn [15] for the development of personal experiential statements and group experiential statements from notes and comments extracted from transcripts, which were then linked across multiple transcripts and grouped into group experiential statements. The researchers used quotations from participant interviews to provide context for their interpretations and demonstrate the dependability of the analysis.

### Positionality

Researchers recognize the importance of positionality in qualitative research, particularly within IPA. Bracketing is a key practice in IPA, where researchers intentionally set aside their preconceived notions and biases to better understand the participants'experiences [16]. The positionality of the first author, an Indian cisgender man aged 26 who works as an Assistant Professor, and the second author, an Indian cisgender woman aged 58 who works as a Professor, is acknowledged. This recognition highlights the potential influence of their cultural backgrounds and gender perspectives on the research. By engaging in bracketing, the authors actively strive to minimize these influences, ensuring a more authentic representation of the participants'lived experiences while remaining conscious of their positionality.

### Participants

For the study, participants were required to self-identify as cisgender males living in India. When selecting research participants, it is crucial to determine the appropriate sample size, as suggested by Creswell [17], who stated that"it is important to determine the size of the sample you will need"(p. 146). For phenomenological research, the participant pool can range from 2—25. Clarke [18] also postulated that three is the default sample size for undergraduate or master’s-level IPA studies, whereas 4—10 is advised for professional doctorates. Conducting an IPA research study with homogenous participants provides a better understanding of their lived experiences. The researchers recruited six cisgender males for the study, a small sample size, to examine convergence and divergence in detail [19]. All the participants were over 18 years old; they spoke English fluently, and no translation was needed. None of the participants received any compensation for their involvement. Data were collected from the six participants whose ages ranged from 19–28 years (M = 21, SD = 4.07), and their identities were kept confidential by changing their names (Table 1).

**Table 1:** Presents the names, ages, genders, sexuality, and pronouns of the participants

NAME	AGE	GENDER	SEXUALITY	PRONOUNS
Arya	19	cis-gender man	heterosexual	he/him
Harshit	20	cis-gender man	heterosexual	he/him
Sid	19	cis-gender man	heterosexual	he/him
Jeet	19	cis-gender man	heterosexual	he/him
Aswin	26	cis-gender man	heterosexual	he/him
Jerry	28	cis-gender man	heterosexual	he/him

### Materials

After receiving approval from the ethical committee of the School of Social Sciences and Languages at Vellore Institute of Technology, Chennai, India, for human participant research, a survey asking for consent to participate in the interview was circulated through social media platforms such as WhatsApp groups and Instagram. Participation was requested exclusively from individuals who identified as a cisgender male. Men who expressed interest in participating in the research study provided their consent by completing and signing a form granting permission for the interview. The researchers formulated ten open-ended questions [20] without direct connection to literature or theory (refer to Table 2).

**Table 2:** Outlines the open-ended questions asked in the interviews

Q. No	QUESTIONS
1	How do societal expectations of masculinity influence your perception of your body?
2	Can you describe any subjective experiences where you felt pressure to conform to certain physical ideals associated with being a man?
3	In what ways do you think media representations contribute to shaping male body image perceptions in our society?
4	Have you personally experienced body dissatisfaction or concerns related to muscle dysmorphia?
5	How do you think cultural norms in India impact the way men view their bodies compared to other cultures?
6	Have you ever felt judged or stigmatized based on your physical appearance, particularly concerning your masculinity?
7	Do you think there is enough awareness and support available for men who struggle with body image issues in our society?
8	How do you think traditional notions of masculinity affect men's mental health, particularly about body image concerns?
9	What do you believe are the most effective strategies for promoting body positivity and challenging harmful stereotypes about male bodies?
10	From your perspective, what policy changes or societal shifts could help address the gaps in support for men dealing with body image issues?

### Procedure

The study involved individual interviews conducted in person in designated discussion rooms and online via Zoom, interviewed by the first author. These semi-structured interviews lasted between 35 and 56 min (M = 37.47, SD = 9.16). The semi-structured format facilitated researcher‒participant engagement, allowing flexibility in the interview questions and order based on participants'responses [15, 21]. The participants were free to withdraw from the voluntary study anytime for comfort. All interviews were recorded with participants'consent via the Voice Memos app on an iPhone and Zoom and were transcribed later via the paid online tool, Turboscript.

### Epistemological and ontological position

The study adopts a constructivist epistemological stance, recognizing that knowledge is co-constructed through the interaction between the researcher and the participants. This approach acknowledges the subjective nature of reality, where experiences are shaped by individual perceptions and social contexts. Ontologically, the research aligns with a relativist perspective, which posits that multiple, context-dependent realities exist.

These epistemological and ontological positions are congruent with the IPA's focus on understanding the lived experiences of individuals. IPA emphasizes the subjective meaning-making processes and acknowledges that both the researcher’s interpretations and the participants'accounts contribute to the understanding of the phenomenon. This analytical approach seeks to explore how participants make sense of their body image experiences in light of cultural and societal influences, rather than seeking a singular, objective reality.

### Transparency and trustworthiness

To ensure the transparency and credibility of the analysis, the researchers adhered to the guidelines outlined by Smith [19]. The research process was meticulously documented, allowing for an audit trail that others could follow to understand how interpretations were derived from the data. The credibility of the findings was further enhanced through member checking, where participants were invited to review and provide feedback on the preliminary experiential statements generated from their interviews. The member-checking process revealed that participants felt their experiences were accurately represented, with no requests for changes to their interviews. This outcome indicates a high level of agreement and validation regarding the experiential statements, and interpretations presented in the study. Additionally, the researchers engaged in peer debriefing sessions to critically examine group experiential statements and challenge potential biases.

### Data saturation and development of the interview schedule

The study defines data saturation as the point at which no new experiential statements or insights emerge from the data, indicating that the dataset is comprehensive enough to address the research question. While the application of saturation in IPA is debated, the researchers took a pragmatic approach aligned with ensuring a thorough understanding of the phenomenon while remaining cautious of overextending this concept within the IPA framework. The interview schedule was developed iteratively, with open-ended questions refined on the basis of pilot interviews and expert feedback to align with the phenomenological aims of the study, maintaining a balance between structure and flexibility to explore participants'unique perspectives.

### Data analysis

#### Interpretative phenomenological analysis

Although 10 open-ended questions were posed to participants, only responses directly relevant to the study’s research question were included in the analysis. Tangential discussions or off-topic narratives were excluded to maintain the focus on body image experiences, societal influences, and cultural norms. The analysis involved a detailed examination of the transcripts, following the process outlined by Smith and Osborn [15]. Each transcript was carefully read to note comments, such as summaries, paraphrasing, associations, and preliminary interpretations in the margin. After the initial read-through, common experiential statements were identified from the notes and compared with group experiential statements from other transcripts. The accuracy of the experiential statements capturing the participants'experiences and interpretations was double-checked for each transcript. The semi-structured nature of the interviews allowed for the intentional inclusion of questions on the group experiential statements in later interviews to achieve saturation. The initial experiential statements were then renamed in an interpretive manner with the assistance of the second author. The second author was consulted again to assess the application of quotes to the newly refocused and renamed experiential statements (refer to Table 3), resulting in a stronger focus [15, 22, 23]. "Being a male in India"is a group experiential statement, from which other statements emerge. Currently, for participants, this experience fosters an “The'I Have To'Mentality”. This mindset encourages constant “Body Surveillance” and cultivates “Feelings of Inadequacy” when their appearances do not align with societal ideals, ultimately leading to “Body Dysmorphia”.

**Table 3:** Outlines the group and personal experiential statements discussed in the results, providing an overview of the issues faced by the participants in relation to body image

Experiential Statement Type	Statement Label	Description
GROUP EXPERIENTIAL STATEMENT 1	BEING A MAN IN INDIA	The cultural context of India imposes pressures related to traditional masculinity, media influence, and a lack of support for men's body image concerns
*Personal experiential statement 1.1*	*The'I Have To'Mentality*	Internalized expectations drive men to conform to societal ideals of masculinity and appearance
GROUP EXPERIENTIAL STATEMENT 2	OBJECTIFIED BODY CONSCIOUSNESS	How participants experience their bodies as objects to be evaluated against societal standards rather than as integral aspects of their identity
*Personal experiential statement 2.1*	*Body Surveillance*	Participants constantly monitor their physical appearance due to societal expectations and comparisons with idealized body standards
*Personal experiential statement 2.2*	*Feelings of Inadequacy*	Many experience shame and low self-esteem when they feel they do not meet the ideal male body standard
GROUP EXPERIENTIAL STATEMENT 3	BODY DISSATISFACTION	The culmination of internalized pressures and objectified consciousness results in active dissatisfaction with physical appearance
*Personal experiential statement 3.1*	*Body Dysmorphia is Real*	Distorted body image perceptions lead to struggles in reconciling actual appearance with internalized ideals, often resulting in psychological distress

## Results

### Being a man in India

#### Primary group experiential statement

The conversation surrounding male body image in India sheds light on the escalating acknowledgment of the societal pressures and unrealistic standards that men encounter, as well as the necessity for addressing these issues more openly. The participants in the dialogue underscored several crucial areas requiring change, indicating a longing for a more supportive and inclusive environment.

Arya brought attention to the contrast between India's approach to male body image and the more progressive attitudes prevalent in Western cultures. He noted that in countries like the U.S. and the U.K., body positivity and men's health are frequently discussed topics, especially on social media. Influencers in these regions openly tackle issues such as body dysmorphia and mental health, fostering a supportive community that is largely absent in India. Arya stressed the need for similar discussions within the Indian context, acknowledging the potential of social media and influencers in propelling this change.

His observations indicate that India is at an early stage of developing a discussion around male body image. He encourages greater visibility and support in this area. By promoting body positivity and raising awareness about mental health, Indian influencers, and content creators could contribute to creating a more inclusive and supportive online environment. This could encourage men to openly discuss and address their body image concerns.

Aswin highlights the lack of support groups in India, particularly in comparison to their portrayal in Hollywood films. He points out that in Western media, support groups are a common resource for individuals dealing with mental health challenges, offering a space to share experiences and find comfort in community. However, such spaces are rare in India, even within educational institutions. Aswin recommends that universities and colleges could be ideal starting points for establishing these much-needed support systems.

The participants’ suggestions underscore the importance of educational institutions in fostering mental health support. By creating inclusive support groups that welcome individuals of all genders, universities can play a pivotal role in promoting open dialog and peer support. This approach highlights the therapeutic benefits of sharing experiences in a nonjudgmental environment and the need to address the diverse mental health needs of students.

Harshit sees mass sporting events such as the IPL (Indian Premier League) as powerful platforms for promoting inclusivity, particularly for marginalized communities such as the LGBTQ+ community. He suggested that strategic advertising and advocacy during these high-profile events could significantly impact societal attitudes and norms. Harshit noted that the IPL's broad and diverse view makes it an ideal venue for spreading messages of inclusivity and raising awareness about important social issues.

His perspective highlights the role of mainstream media and culturally significant events in shaping societal attitudes. By leveraging the visibility of events such as the IPL, advocates can reach a wide audience and promote messages of inclusivity and acceptance. This approach emphasizes the importance of representation and visibility in building a more inclusive society, particularly for marginalized groups.

Jeremy emphasizes the necessity for more balanced health policies in India, particularly those that address men's health concerns. He criticizes the current emphasis on women's health in government initiatives, arguing that men's health needs should also be given priority. Jeremy also highlights the impact of social media and societal standards on health decisions, noting that these often prioritize superficial objectives over overall well-being. He calls for clearer guidance and improved policies that take into account the health needs of all genders and promote comprehensive health and well-being. His reflections underscore the challenges of making health decisions in a society influenced by conflicting information and societal pressures. He stresses the importance of evidence-based guidance and balanced health policies that support the holistic health of all individuals, challenging societal norms that prioritize appearance over genuine health.

### The “I have to” mentality

#### Personal experiential statement derived from being a man in India

The recurring personal experiential statement of "I have to" in the participants'narratives vividly illustrates the internalized pressures and societal expectations that shape their self-perceptions and behaviors. This phrase, consistently repeated across their accounts, reflects a profound sense of obligation driven by external influences such as societal norms and media portrayals, revealing how deeply these pressures are embedded in their daily lives.

Arya’s narrative is a clear example of this compulsion. His statement, "I mean, I have to start somewhere," signals a perceived necessity to embark on a journey toward achieving an ideal body image, despite the mental and emotional toll it takes on him. His avoidance of mirrors at the gym, as he explains, "I tend to stay away from the mirrors… I have to, I cannot mentally size up to them," exposes the psychological burden he carries in trying to measure up to the physiques of others. Arya's repeated phrases, such as "I have to eat more calories" and "I have to maintain a certain amount of weight", underscore how societal standards have infiltrated his mindset, compelling him to continually strive toward an often unattainable ideal.

Similarly, Harshit articulates the weight of societal expectations with his frequent use of "I have to. " His statement, "I have to reduce my weight," reflects the external pressures dictating his body image goals, whereas "I also have to take, you know, my surroundings into consideration," reveals his awareness of how others perceive him. This sense of obligation is pervasive in his actions, as he mentions, "I have to again stop eating or again have to go on a diet," and "I have to look good to impress." Harshit's narrative highlights a relentless struggle to align his physical appearance with societal ideals, driven by a desire for acceptance and approval that overrides his personal preferences and well-being.

Jerry's account reflects the experiential statements of compulsion, particularly in his admission that "I have to follow them consciously or subconsciously or somehow. "This suggests that societal pressures influence his behavior, even when he is not fully aware of it. The expectation to maintain physical fitness is evident in his statement, "I have to work out more," reflecting the internalized belief that he must conform to an idealized body image. Jerry further elaborates on this burden, noting, "Society indirectly expects or mentions that men have to be strong physically, and' just because you are a man, you have to tolerate everything.' "This reveals the heavy standards imposed on men to embody strength and resilience. Moreover, his remarks, "I have to impress others," and "I want to impress others because I do not live for myself," expose a life driven by the need for external validation. This need often leads to a disconnection from personal fulfillment, illustrating the deep conflict between individual desires and societal demands.

Sid's narrative poignantly captures the experiential statements of feeling compelled through his struggle with societal expectations to embody a certain degree of physical and emotional rigidity. His statement, "I have to be, like, rigid, like, stuffed, beefed," encapsulates the pressure to conform to an idealized image of masculinity. The use of "stuffed" suggests a sense of being overfilled or burdened, indicating that the effort to meet these standards is overwhelming and unnatural. The repetition of "like" highlights the performative aspect of this identity, suggesting that these attributes are not inherent but rather imposed by societal norms.

In each of these narratives, the phrase "I have to" serves as a powerful reminder of how societal expectations shape men's lives, often leading to a constant struggle to meet external standards. This compulsion to conform can result in significant emotional and psychological strain, as these men navigate the tension between their desires and the relentless demands of societal ideals.

### Objectified body consciousness

#### Group experiential statement emerging from the "I Have To" mentality

The "I Have To" mentality directly contributes to an objectified body consciousness among the participants. This group experiential statement encompasses how participants view their bodies as objects to be evaluated against societal standards rather than as integral aspects of their identity. It manifests through two distinct personal experiential statements: Body Surveillance and Feelings of Inadequacy.

### Body surveillance

#### Personal experiential statement under objectified body consciousness

The men reported frequently engaging in a behavior known as "body monitoring" or "self-surveillance," where they maintain a persistent, often subconscious awareness of their physical appearance. This heightened self-awareness is not only about maintaining personal standards but also about being mindful of how others perceive them. It is evident in frequent mirror checks, adjustments to posture, and deliberate clothing choices aimed at downplaying perceived flaws. This heightened self-awareness can often lead to discomfort and dissatisfaction with their bodies, as men strive to meet societal expectations that are often unattainable.

Arya, for example, discusses the pressure to conform to a muscular, gym-centric body image, a standard that is constantly reinforced by social media. He describes the feelings of inadequacy that arise when he compares himself to others, particularly in environments that emphasize physicality, such as gyms or elevators. Arya's experience highlights how exposure to more muscular physiques can trigger body dysmorphia, a condition that makes it difficult for him to reconcile his self-image with the ideals he has internalized. He explains:

*When I see somebody truly tall, I just instinctively have the urge to try and height up there... Another would be I'm in the gym, right? I tend to avoid looking at the mirrors because then you see many of the other people who are working out there... In addition, so you look back at your own hands and you look at your own body, and you get like a bit of body dysmorphia.*

This example illustrates how social comparison, fueled by pervasive media representations, exacerbates Arya's struggle with body image, leading to ongoing self-surveillance and dissatisfaction.

Harshit's journey reflects the societal pressures associated with the "healthy male body" ideal, leading to feelings of guilt when he gained weight. This guilt was compounded by subtle expectations from his father, who, although not overtly critical, still motivated Harshit to lose weight. His story unveils the constant struggle between self-acceptance and societal demands for a specific physical appearance. Harshit acknowledges that, while at times he gives in to "laziness," the pressure to conform to societal standards remains a persistent aspect of his life.

Aswin's experience with body monitoring is centered on his discomfort with his "bigger thighs," a feature that complicates finding clothes that fit properly. He noted how societal expectations and fashion trends intensify this discomfort, frustrating him with his inability to "adapt to new trends" due to these physical limitations. Despite his awareness of media influence on body image, Aswin struggles with dissatisfaction, revealing the deep-seated impact of societal ideals on personal body perception.

Jeet's narrative highlights the pressure to maintain a certain body fat percentage, driven by external critiques, particularly from acquaintances at the gym. His experience reveals a cycle of body surveillance, where he is both the observer and the observed, driven by a desire to conform to an ideal physique. This pursuit, often exacerbated by media portrayals and peer comparisons, leads to an internal conflict that underscores the psychological toll of these societal pressures.

Jerry's story further emphasizes the external pressures men face, particularly the unspoken societal expectations of being physically strong and protective. He expressed a deep fear of failing to meet these standards, fearing potential damage to his reputation:

*They expect me to be strong. They expect me to protect them whenever there is an issue... I don’t like this unwanted attention because I fear the day when someone pushes me down.*

This constant scrutiny, coupled with the pressure to conform to an idealized body image, contributes to a sense of vulnerability and fear of failing to meet societal expectations. Jerry also acknowledges the role of media in normalizing these pressures, highlighting how pervasive and influential these portrayals are in shaping unrealistic body ideals.

Sid echoes similar sentiments, articulating how societal expectations of masculinity influence his perception of his body. He acknowledges that this internalized scrutiny extends to casual interactions, where body-shaming can occur even among friends:

*I need to be big... just to impress society. When we are hanging out with friends, they make fun of, you know, how you look.*

Sid's account reveals how deeply ingrained these body image concerns are, illustrating how they affect not only personal behavior but also social interactions and mental well-being.

These narratives collectively illustrate the pervasive impact of body monitoring on men, driven by external pressures and the desire to conform to a particular masculine ideal. The ongoing self-surveillance and internal conflict these men experience underscores the profound and often damaging effects of societal expectations on body image and mental health.

### Feelings of inadequacy

#### Personal experiential statement under objectified body consciousness

The emotional toll of surveillance on men is profound and deeply ingrained, with participants describing feelings of shame, embarrassment, and inadequacy when they compare themselves to the "ideal" male body. This ideal, often depicted as muscular, lean, and physically dominant, becomes a benchmark against which they measure their worth, leading to episodes of low mood, anxiety, and, in some cases, depression. The impact of this pressure is not fleeting but rather a persistent burden that shapes their self-perception and daily experiences.

The experiences of Arya and Harshit exemplify the profound emotional connection between body image, societal expectations, and personal identity. Arya reflects on the constant pressure to conform to an idealized version of masculinity, expressing,"I always felt like I had to be more muscular, more fit, because that is what men are supposed to be."This societal expectation to embody a specific physical form leads to feelings of inadequacy and a persistent sense of falling short, as Arya confesses,"No matter how much I worked out, I never felt like I was enough."His narrative reveals a significant internal conflict between the desire for personal acceptance and the overwhelming influence of societal ideals. This struggle shapes their self-worth, making it dependent on external validation rather than intrinsic self-esteem.

Harshit’s story further illustrates the relentless nature of these pressures. Despite his substantial efforts to improve his health and body image, including a structured weight loss journey guided by a nutritionist, he continues to grapple with daily dissatisfaction, particularly with his"tummy,"which remains a focal point of his self-consciousness. This ongoing struggle underscores the profound emotional impact of striving to conform to societal standards. Even personal achievements in health and fitness are overshadowed by the relentless pursuit of an elusive ideal. Harshit’s experience highlights the exhausting and demoralizing nature of these pressures, revealing how they can diminish the satisfaction that should come from personal accomplishments. Instead, the constant awareness of falling short of societal ideals continues to exert a significant emotional burden, perpetuating feelings of inadequacy.

Sid’s narrative delves into the pervasive inferiority complex driven by pressure to"impress society"and conform to the ideals of being"big"and physically imposing. He describes how even casual remarks from friends, such as"I'm not hurting you only because I know you cannot hurt me back,"leave lasting scars, reinforcing his sense of inadequacy. These seemingly offhand comments contribute to deep emotional strain, exacerbated by the societal expectation that men should not be vulnerable. Sid reflects on the cultural dictum that"You're a man, men do not cry,"which highlights the pressure to suppress emotions and conform to a rigid and narrow ideal of masculinity. This expectation not only forces him to hide his true feelings but also leads to an ongoing internal conflict, leaving him feeling disconnected from his authentic self. The need to constantly present a tough, unfeeling exterior takes a toll on mental health, creating a cycle of emotional repression and dissatisfaction.

Collectively, these narratives reveal the profound and enduring emotional impact of sexual objectification and societal pressure on men. The internalized expectations to embody an idealized form of masculinity create a complex and often painful struggle with self-worth. These pressures, rooted in societal norms and media representations, lead to a persistent sense of inadequacy, anxiety, and depression, as men navigate the tension between who they are and who they feel they must be. The emotional burden of striving to meet these ideals underscores the need for broader societal conversations about masculinity, body image, and the importance of self-acceptance.

### Body dissatisfaction

#### Group experiential statement emerging from the "I Have To" mentality

The third major group experiential statement that emerges from the "I Have To" mentality is Body Dissatisfaction. This represents the culmination of internalized pressures and objectified body consciousness, manifesting as active dissatisfaction with one's physical appearance and, in more extreme cases, body dysmorphia.

### Body dysmorphia is real

#### Personal experiential statement under body dissatisfaction

Arya's narrative offers a poignant and deeply personal portrayal of how body dysmorphia shapes his daily life, particularly in the context of his experiences at the gym. He depicts the temporary "pump"— the swelling of his muscles during workouts — as a brief moment of gratification, during which he feels strong, powerful, and in harmony with the idealized male physique. However, this sense of empowerment is short-lived. Arya reflects on how the strategic lighting and mirrors at the gym intensify this illusion, resulting in a distorted self-image that quickly fades once he leaves the gym. At home, under harsher lighting, the pumped-up version of himself diminishes, and he is confronted with a more familiar, less idealized reflection.

Arya's statement, "Body dysmorphia is like a daily thing for me now," underscores the enduring nature of this condition. He describes a painful cycle of self-perception, where the temporary euphoria of seeing his pumped muscles gives way to intense disappointment when the effect dissipates. The gym, intended to be a place of self-improvement, becomes a battlefield for his mental and emotional well-being, where he continually struggles with the contrast between his pumped-up physique and his everyday appearance. This cycle worsens his dysmorphia, leading to an unrelenting effort to reconcile his self-image with the fleeting, idealized version he sees during workouts.

He also delves into the societal pressures that shape his self-perception, particularly the influence of gender norms. He recounts being criticized for his hairstyle and facial hair, which did not conform to traditional masculine standards. This pressure led him to alter his appearance, not out of personal preference, but to avoid negative feedback from others. In his reflections,"When I had long hair with a middle part, people said I looked like a girl. In addition, my mustache doesn’t grow thick—it’s thin and patchy,"reveals the deep emotional burden of navigating these external pressures. Aryas’ willingness to change their appearance to avoid criticism highlights the profound impact of societal expectations on individual self-worth, especially when those expectations are tied to rigid gender norms.

Aswin’s personal experiences with clothing challenges serve as a poignant illustration of the difficulties faced by individuals whose bodies do not align with conventional standards. His frustration in finding well-fitting pants sheds light on the systemic issues surrounding size inclusivity and the fashion industry’s limited definitions of acceptable body shapes. Aswin's recourse to tailoring his clothes underscores his desire for autonomy over his appearance, emphasizing the importance of personal agency in a world that often disregards nonstandard bodies. His story highlights the significance of individual choice in clothing and the mental toll of an industry that marginalizes those outside its narrow size spectrum.

Furthermore, Aswin delves into the cultural associations between body size and gender, observing how smaller male bodies are often feminized or seen as lacking masculinity. He reflects on how literature and cultural representations perpetuate these stereotypes, perpetuating a cycle of insecurity for those who do not conform to traditional standards of masculinity. His observation that"Smaller bodies are usually associated with women. If a man has a smaller frame, it is often perceived as a deficiency as if he is less masculine"exposes the cultural roots of body dysmorphia and sheds light on how societal norms about masculinity and body size can lead to feelings of inadequacy. Aswin’s insights underscore the importance of critically examining and challenging these narratives, which profoundly impact self-esteem and identity.

Harshit shares his experience of social anxiety related to body image, particularly the impact of societal expectations on mental health. He discusses how social media can exacerbate insecurities and how he has managed to avoid some of these pressures by distancing himself from these platforms. His reflection, "It’s embarrassing and anxiety-inducing to be in social situations, especially as a teenager with things like pimples," highlights the pervasive nature of body dysmorphia and its intersection with social anxiety. By removing himself from social media, Harshit mitigates some of the pressure, but his experience illustrates how societal standards still infiltrate everyday life, leading to ongoing struggles with self-image.

This study provides an analysis of how steroid use and misinformation in the bodybuilding community can contribute to body dysmorphia. He criticized the lack of transparency among Indian bodybuilders regarding steroid use, which can lead to unrealistic expectations and further exacerbate body image issues. In Jeet’s account,"Indian bodybuilders aren’t open about steroid abuse. When they post their pictures, people think that’s naturally achievable, and they keep working out, chasing something that isn’t real,"sheds light on the dangerous impact of misinformation. His insights emphasize the importance of promoting body positivity and accurate information within the fitness community to prevent the psychological toll of striving for unattainable ideals.

Sid reflects the psychological effects of body dissatisfaction, particularly how it affects individuals’ sense of agency and their ability to respond to threats. He describes how body dissatisfaction can lead to a feeling of helplessness, where the fear of being unable to defend oneself is tied to negative self-perception. Sid’s narrative reveals the deeper psychological implications of body dissatisfaction, extending beyond appearance to influence how individuals perceive their strength and autonomy. His reflection, "If someone attacks me and I can’t defend myself because of my body, that’s where dissatisfaction truly hits. It’s not just about looks; it’s about feeling powerless," emphasizes the importance of promoting positive body image and self-esteem, not only for mental well-being but also for fostering a sense of empowerment and resilience in challenging situations.

These narratives collectively highlight the pervasive and damaging effects of societal pressures on body image, self-esteem, and mental health. Each participant's experience underscores the need for a broader societal shift toward accepting diverse body types and challenging rigid gender norms. The emotional toll of body dysmorphia and the pressures to conform to idealized standards of masculinity reveal a profound struggle that affects not only physical appearance but also personal identity, self-worth, and psychological resilience.

## Discussion

This study sought to answer the research question: *What specific experiences and challenges concerning body dissatisfaction, muscle dysmorphia, and the pursuit of muscularity ideals are identified by a sample of men living in India?* By examining participants'narratives through IPA, the findings shed light on the complex interplay of societal expectations, cultural norms, and psychological challenges that shape male body image in the Indian context.

### Problematizing societal expectations and masculinity norms

The participants’ narratives reveal deeply entrenched societal expectations that valorize muscularity, physical strength, and stoicism as markers of masculinity. These findings align with prior studies, such as those by Suffolk [24] and Murray et al. [25], highlighting the pressure to conform to rigid gender roles. However, unlike Western societies where body positivity movements are gradually reshaping norms, India’s sociocultural landscape offers limited accommodation for body diversity.

This context challenges the universality of body image theories like objectification theory, which predominantly stem from Western-centric frameworks. While the findings corroborate certain elements of objectification theory [26]—such as the internalization of societal and media-driven ideals—their applicability in non-Western settings remains limited. In India, traditional gender norms coexist with modern body ideals, creating unique pressures that compound the experiences of body dissatisfaction and self-surveillance among men.

### Media influence and cultural norms

Global media portrayals of the ideal male body often depict muscularity and leanness as aspirational standards. The participants’ accounts, such as discomfort in gym environments, underscore how these portrayals exacerbate body dissatisfaction through relentless comparison. This aligns with Labre’s [2] assertion that media-driven ideals disproportionately affect male body image and with Gültzow et al.’s [27] findings on social media’s role in amplifying these standards.

However, the impact of media in the Indian context is magnified by cultural norms that celebrate physical strength as a symbol of status and masculinity. These findings emphasize the need for body image theories that integrate both global and local influences, offering a more comprehensive understanding of how men internalize ideals in culturally distinct ways.

### Perfectionism and the pursuit of the 'ideal'

Perfectionism emerged as a key experiential statement, with participants expressing an internalized obligation to achieve a “perfect” body. For example, one of the participants’ recurring statements about dieting and weight loss highlights the pervasive nature of these pressures. Existing research by Goldfield et al. [7] and Zimik [13] has similarly noted the psychological toll of striving for perfection, linking body dissatisfaction to anxiety, depression, and low self-esteem.

In the Indian context, perfectionism appears to be shaped by individual aspirations and collective societal values. The findings suggest that existing psychological models of body image should be expanded to account for the influence of cultural and communal expectations, which often intensify the pursuit of unattainable standards.

### Psychological impacts beyond body dissatisfaction

The findings extend beyond body dissatisfaction to reveal broader psychological effects, including anxiety, shame, and social withdrawal. Participants described how body image concerns are intertwined with their sense of self-worth, social interactions, and emotional well-being. These findings challenge the sufficiency of focusing solely on body dissatisfaction in body image research. Instead, they call for an integrated approach that considers the intersection of body image with mental health and social identity, particularly in societies where stigma around vulnerability may amplify these struggles.

### Gaps in support systems and cultural awareness

A critical issue highlighted by participants is the lack of support systems for men struggling with body image issues in India. One of the participants’ reflections on the absence of support groups resonates with Soohinda et al.’s [14] findings on the limited awareness and resources available for addressing male body dissatisfaction. Unlike Western contexts where support structures and mental health resources are more accessible, Indian men often navigate these challenges in isolation.

This gap underscores the need for culturally sensitive interventions that address the unique sociocultural dynamics influencing body image in India. For instance, promoting body positivity through public campaigns, integrating mental health support within educational institutions, and leveraging popular platforms like the Indian Premier League for advocacy could create a more supportive environment.

### Limitations and future directions

Overall, the study underscores the limitations of applying existing body image theories developed in Western contexts without adaptation. The findings suggest a need to revise theoretical models to better accommodate the experiences of men in cultures where masculinity is defined differently. Future research should focus on creating more inclusive frameworks that incorporate diverse cultural perspectives, while also examining intersectional factors such as caste, religion, and socioeconomic status. Additionally, there is a pressing need for interventions that challenge harmful stereotypes and promote body positivity in ways that resonate within the cultural context of India.

The results of the research are specific to men living in urban areas, which may limit the transferability of the results. This focus on urban participants may not fully capture the diversity of experiences related to body image across different regions and socioeconomic backgrounds in India.

Another limitation concerns the homogeneity of gender identity and sexual orientation within the sample. All participants identified as cisgender heterosexual men, which excludes the experiences of individuals with diverse sexual orientations or gender identities. This homogeneity limits the study's ability to explore how intersections of gender identity, sexual orientation, and body image might manifest differently across the spectrum of masculinities in India. Future research should aim to include participants with diverse sexual orientations and gender identities to develop a more comprehensive understanding of body image concerns beyond the cisgender heterosexual experience.

The reliance on self-reported data introduces the possibility of social desirability bias, as participants may have underreported or exaggerated certain experiences due to the stigma associated with discussing body image concerns among men. Although efforts were made to create a comfortable interview environment, the sensitive nature of the topic could have affected the depth of disclosure.

The study's qualitative design, while appropriate for exploring lived experiences, does not allow for the identification of causal relationships or the prevalence of specific body image issues. The findings offer a rich understanding of individual experiences but should be viewed as a starting point for further research rather than a comprehensive account of all aspects of male body image in India.

The cross-sectional nature of the study captures participants'experiences at a single point in time, limiting the ability to explore changes in body image perceptions over time. Longitudinal studies could provide a more dynamic understanding of how body image concerns evolve in response to shifting cultural norms or life events.

### Implications

Despite these limitations, the study has important implications for both research and practice. The findings highlight the need for more culturally sensitive approaches to studying body image, particularly in non-Western contexts where traditional gender roles and modern ideals often coexist in complex ways. Researchers should consider developing theoretical models that incorporate cultural and societal factors unique to the Indian context, moving beyond frameworks primarily based on Western experiences.

For practitioners, there is a clear need to design interventions that address the specific pressures faced by men in India, such as the expectations to embody traditional masculinity while conforming to modern body ideals. Mental health professionals and educators should be aware of the cultural stigma surrounding body image issues in men and work to create safe spaces where these concerns can be openly discussed without judgment.

Furthermore, the lack of accessible support systems for men struggling with body image in India underscores the importance of policy efforts aimed at expanding mental health resources and awareness. Interventions that include media literacy programs to counteract unrealistic body ideals and promote diverse representations of masculinity could help mitigate the negative impacts of societal pressures.

Overall, this study serves as a foundation for future research and practice aimed at understanding and addressing male body image concerns within specific cultural contexts, emphasizing the importance of tailored approaches that reflect the lived realities of men in India.

## Supplementary Information

Supplementary Material 1.

Supplementary Material 2.

Supplementary Material 3.

Supplementary Material 4.

Supplementary Material 5.

Supplementary Material 6.

## Data Availability

The original contributions presented in the study such as interview transcripts have been deposited as supporting documents in the "supplementary files" for the journal to review. Further inquiries can be directed to the corresponding author for the reliability and validity of the research. The raw de-identified data may be made available upon reasonable request from the corresponding authors.

## Abbreviations

IPA: Interpretative Phenomenological Analysis
BID: Body Image Dissatisfaction
BMI: Body Mass Index
