# Supplementary material for: “An interpretative phenomenological analysis of male body image through the lived experiences of men in India”
Source: BMC Psychol. 2025 Jul 1;13:714. doi: 10.1186/s40359-025-02963-y (PMC12219639; doi:10.1186/s40359-025-02963-y)
Supplement: Supplementary file 1 — Supplementary Material 1. [file 40359_2025_2963_MOESM1_ESM.pdf]

(0:03 - 0:15)

Okay, Mr. *****, good morning. Morning, sir. Okay, do you know about this study a little bit? Because it showed interest.

(0:17 - 1:01)

Yes, yes. I mean, if you could talk about it, it would be enlightening. Okay, sure.

Okay. So, the name of the study is an interpretative phenomenological analysis of male body image through the lived experiences of men in India, bridging policy and practice for gender equality and sexual diversity. Okay.

So, you know how research is done, right? Yes, yes. I mean, Yeah, do you know the research process we have? Do you know any idea like? So, you collect data, then, you know, analyze the data, then come up with your interpretation. Yeah, that's what we call, you know, methodology methods, you know, qualitative or quantitative, then we have methods, right? So, we have interpretative phenomenological analysis.

(1:01 - 1:48)

It's a method. Okay, it's a qualitative method. So, basically, we interview people.

Okay. And we analyze it. Okay.

So, when we say interpretative phenomenological analysis, it's basically the lived experiences of the people. Okay, so, you already interpret the, you know, existing phenomena, it already happened to you. So, it's already there.

And you are interpreting to me, okay, and I am interpreting for you. Okay, in the paper.

So, that's how this method works.

Okay. Okay. So, so we are using the concept of male body image, any idea what male body image encompasses? I think body image means how people feel about their bodies.

(1:51 - 2:10)

Okay. Like in what terms, in what terms like any idea? I'm not really sure about those parameters. But I think body image is about, generally is about how you feel about yourself, your body, the way it is, can be, you know, positive or negative.

(2:11 - 3:52)

Okay. Okay. So, let me ask you a few questions.

I'll get to start. Okay. So, how do societal expectations of masculinity influence your perception of your body? Should I answer in a general sense or personally? You can ask, I mean, you can answer in general perspective, as well as, you know, your personal from your personal.

Yeah. So, I think, the societal expectations of masculinity have been there for centuries together. I think in the Jewish tradition, maintaining a beard meant masculinity.

So, we have, we've had several forms of, you know, this, this trend continue until this age. So, I think a lot of people who cannot grow beards, feel inferior, feel less manly, due to their inability of growing a beard. I think this is a real phenomenon.

So, I think there's one example that I can point out regarding societal expectations of masculinity, and moustache as well. So, I think that is reinforced in our Indian culture. I mean, having a moustache is, you know, equated to manliness, royalty, or things like that.

So, people who are unable to grow facial hair, probably feel, I mean, they talk about it often, I'm not sure if they're insecure about it, but they talk about it often. I'm not able to grow a beard, I'm not able to grow a moustache. When will this grow? There are such concerns among youngsters and other people.

(3:53 - 5:20)

So, can you relate it to your body image? How does this, you know, how do these expectations influence your perceptions of your body? So, I think growing a beard was a taboo, I guess. I think at least in some of our homes, I think it was a taboo. Okay, it is associated to people being romantic failures or such, means it's generally connected to such people, right? People who are artisans, people who have failed in their drunkards and things like that.

But then, recently, due to modern films and all that have captured beard as something that is an essential thing to have, something that's positive, something that is admired by everybody. So, it has had a rather, I mean, people do not complain about having a beard. I mean, earlier, when I used to grow my beard, I was often asked to, you know, take it off.

It doesn't look good. It is not something that good people grow or things like that. But then now, it has become very normalized.

Growing a beard can be short, long or whatever. And I feel like the people around me have started complaining less about having it. It's sort of normalized.

People don't ask about it in a curious sense. Okay, why are you growing a beard? They don't ask that because their own children probably have started doing that. So, it's normal.

(5:20 - 5:51)

So, I think that's a personal experience. Okay, that's interesting. Anything else about, okay, you talked about all this, you know, facial hair and stuff.

Anything about, you know, our, you know, maybe our physical body except our facial hair? Yeah, okay. So, I have big thighs. Compared to the average person, I have bigger thighs.

(5:51 - 6:33)

So, I'm not really comfortable with that because I don't get pants my size. You know, even if my waist, even if I get pants that are, you know, close to my waist size, let's say 36 inches or 38 inches, my thighs don't fit in. So, I'll have to get an additional size like 40 or something for my thighs to accommodate my thighs.

So, this is not, this is something that I'm not really comfortable with only because of this reason. And even when I try to lose weight, the fat in thighs, thigh fat or I don't know if it's the structure, I don't know how to explain this in medical or biological terms. But I've been rather unsuccessful in trying to reduce the size.

(6:34 - 9:20)

So, this is something that I'm not really comfortable. Why is that? Because, you know, like, so let's relate it to the societal expectations of masculinity. Okay, this is your expectations.

Okay, I don't want this. This has nothing to do with the societal thing. I'm simply annoyed because I don't get the pants which I really want.

That is the only problem. You know, there are, when you go to shop, you really want to,

you know, buy a certain pant. And you see that, okay, we are okay.
I have this also my waist size. Let me try this. Then when I try that, you know, I'm not able to fit in.
So it feels, you know, very, you know, Oh, God, why is it like this? Why don't I get pants my size? Why can't I get things that I really want to do? And I often have to go for stitching. Because when I buy the material and stitch it, I can customize it according to my body shape and things like that. This is something that I am personally annoyed by.
Yeah. Okay. Like, is there anything else? Like, I want to be like this? Because you know, like, it's how people expect.
It's how you know, the people have expectations of me to, you know, to portray myself in a way that they all like, maybe I would conform to the expectations of the society so that I can, you know, dress up like this. So I can grow muscles or something like that. Do you have anything, any influence, any societal influence? Regarding this matter, this pants and that thing or I don't think there are any societal maybe with regards to fashion, okay, like now this, this is trending or this is trending, you probably want to try that out. And when you go to the shop and check that out for yourselves, you feel you feel that okay, this fashion is not not meant for me. Because of this problem, not even I consider that a problem. I'm not able to get adapt to new trends or things that I really want to do because of this accommodation, size accommodation problem.
Understood. Okay. Can you describe any subjective experiences where you felt pressure to conform to certain physical ideals associated with being a man? We're talking about physical ideals.
Let's say like, we have, you know, maybe on Instagram or Facebook, we would have, you know, we would follow all these physical ideals, a bodybuilder or an actor who has a, you know, very good physique. Okay, so I just want to write, like, I feel pressured to become like that person. Yes, something like that.
(9:21 - 12:30)
I've not felt pressured, but I thought was cool. It was cool when I was younger. So in 2008, Ghajini released the Hindi version of Ghajini.

So at that point of time, eight packs was the talk of the town. Wherever you magazines, newspapers, how to develop eight packs, think bitter gourd juice, diet plans and all that. So it was something that we all talked about, okay.

So until then, we hadn't heard only about four packs or six packs, eight packs as well. Is eight packs possible? How can this be achieved? Things like that. So I was dumb enough to go to YouTube and, you know, check these workouts, like six minute workout plan, four minute workout plan.

And then since I was younger, I thought, okay, after six minutes, you'll actually develop those abs. But then that's not the case, you'll have to do it consistently. So I think those were my own expectations.

I don't think people really wanted to. Yeah, it was popular then in 2008 when that film released among our circle, like school students. I'm not sure if adults perceive that in the same sense.

Okay. But like, so what about now when you, you know, look at other body shapes, when you come across, you know, internet, okay. So, okay, let's leave the pressure aside.

Okay. If you look at somebody's physique now, okay, because you know, whenever you go to Facebook or Instagram, it's always this gym trend that is popular, all this gym physique that is popular, okay. So when you look at them, okay, maybe, you know, why? Why not, you know, try that? Why not have that? Because we don't have that.

So that that is also kind of a hidden pressure. Okay, because I don't look like them.

Maybe I would, you know, I would be better or maybe I would be admired if I looked like that, if I adopted to that skin.

To be honest, I've not felt pressure to develop somebody else's body or become like somebody else. But I'll say this, I've always been curious, like when I see people who are extremely huge, I often think about what their diet is like, when I the moment I see them, I look at their, you know, structure, the body size and think about what they eat in a day. Like, you know, wouldn't that be expensive? In order to maintain such a body, I think about these things.

I don't feel pressure to do that. So in what ways do you think media representations

contribute to shaping male body image perceptions in our society? I think it has, I think, I think that's a major influence. That's what I mean.

Because this beard trend, I think after the release of Premam, a lot of people wanted to grow a beard and look like Nivin Pauly and things like that. And if you look at actors who were not maintained a beard until for a very long part in their movie careers, I think now all of them have, you know, got into this trend, Tanjani Kamal Hassan, Vijay. In almost all their films, you find them maintaining a beard.

I don't know if it has something to do with societal expectations of media influence. But I think this is the trend now, maintaining a beard. So we know that idea.

(12:31 - 13:27)

That is how it's working. Okay. I think, again, it is a representation of the media, right? So like, you know, viewers like us, you know, watch their movies and we also adopt, you know, like growing beard.

So it has influence. Okay. So what about maybe your physique? Physique.

Physique, I told you about Kajini, that example. I mean, at that point of time as a child, you know, that really had an influence. Okay.

We had never heard about eight abs. We didn't know it was possible. So at that point of time, yes.

How do we achieve this? Okay. Okay. Have you ever personally experienced body dissatisfaction or concerns related to muscle dysmorphia? What does dysmorphia mean? So let's say the const, like, we feel our body is too small.

(13:27 - 13:58)

Okay. Whenever we see other's body, we experience, you know, I don't have the wanted or desired physique, the muscle. Okay.

But I don't possess one. Right. I haven't felt that way.

But then I've told you about the thighs example, right? I feel it's too big in order to, I don't get what I want in terms of fashion. But then I think it does have an impact, like, you know, this body size and all that. I was talking to a friend recently.

(13:58 - 14:28)

He said, I wanted to buy a particular watch, but it's too big on my wrist. So I need to suggest me, you know, something to expand my wrist. Because I am into minor like, you know, not, I'm not a major bodybuilder or something like that.
But I'm into basic muscle building workout and stuff. So when I was talking to him, he said, I wanted to get this watch, my wrist is too small. How do I improve the size of my wrist? So, but I think it's a real thing.
(14:31 - 19:52)
Muscle dysmorphia is a condition. It's a disorder. Okay, so we have this constant, you know, thought of feeling where we, you know, experience that we are not muscled enough.
Okay. We are not defined. Yeah.
When you mentioned that probably as a child, you certainly want to look like those adults, like huge adults who have these shoulders, arms and all that. So that was a big deal back in school, certainly. Arm wrestling competitions, you know, it should actually look big.
That was a goal back in school. So how do we make it big? So these were, you know, things that we often talk about back in school. Then as an adult, I don't think it has had any effect on me.
You just mentioned that you are working out at home, right? Just home workouts. So do you use dumbbells and stuff? Yeah, dumbbells. Okay.
So yeah, so like you do that occasionally or every day? It's like an everyday routine?
Rarely, rarely. So whenever you do that, whenever you do these dumbbells or any home workouts, so you just, you know, do that and you see the difference, right? Okay. So have you ever felt, okay, this is not enough.
I think we, I need to, you know, like work out more or just to. No, I'll actually tell you why I'm doing that. Because I read somewhere that when your body develops a certain percentage of muscle, muscle starts burning fat.
In order to maintain that muscle, your body starts burning more calories. So I thought it was a good way of, when you develop a certain percentage of muscle, it will also help

you in reducing fat. So the primary goal is to reduce fat, not to develop muscle? To have some sort of, yeah, no, it's to have a physical fitness or things like whatever, however you want to do that.

Okay. So how do you think cultural norms in India impact the way men view their bodies compared to other countries? Cultural norms in India and other countries. Yes.

How do, I mean, yeah. Could you give me an example of some sort so that I can, you know, think of something? Okay. Yeah.

I think I can even infer the example from you. Okay. So when in India, okay.

So when you don't, especially in South, okay. If you don't have this mustache and beard, people will always tease you. I think we would have all experienced that in college.

If we have this enough beard or facial hair, people who do not have that, we always and often tend to tease them, right? People do that. Okay. But in other countries, especially in Western countries, people don't really keep the mustache and beard.

They often shave it. But it's their cultural norm. Okay.

So if, you know, growing beard is kind of, you know, they're not into it. Okay. But we should grow beard.

So that's the difference. Even at homes, you know, if you don't have beard, they will definitely call them, call us girls in India. They'll tease us.

Okay. So that's how, yeah, that's what I mean. How do you think cultural norms in India impact? Yeah.

Cultural norms in India impact? Yeah. The way men view their bodies compared to other countries. True.

I think as you rightly said, there's a, you know, clear-cut distinction in those aspects, like how the culture differs. So in European countries, mustache is generally associated with pedophiles for some reason. There's also a derogatory term called pedo-stache.

So when somebody sports only a mustache, it's called pedo-stache. So I think, since that's deeply ingrained in their society, I don't think anybody would really feel comfortable to maintain a mustache in places where that is common, that trend is like common. So I think, yeah, it certainly has the cultural aspect to it as well.

In India? Well, a beard and mustache is okay. But then mustache alone is generally associated to this aspect. Okay.

In the Western countries. Okay. So how about, you know, in Indian cultural norms, what are the expectations of the people in India? Okay, that is related to culture.

Okay. And not just, we're not only talking about the mustache and beard, it also has something to do with the whole physicality of a person. Okay.

So this is how you should know, have the body, you should not have a tummy. Okay. So see, when I see, you know, the study is on male body image, right? Okay.

Because mostly all the studies, if you look at it, it's always about women. Okay, be it research or be it in any social talks, okay, a topic, it always, you know, falls on women. We always, you know, talk about women bodies.

Okay, why it should be the, you know, talk, okay, topic. Okay. There is not much, there are, there is not much study, or research or even, you know, topic, talk, general talks about male body image.

Okay. Right. So that's why the main objective of the study is all about that.

(19:53 - 25:38)

Okay. So what do you think of the physicalities in our, you know, setting in Indian setting? Okay. So how you view, how I view the body image of men? How does it impact, how the culture impact the, you know, perspective of male body image in India? So I hope you understand.

Yeah, because I think this is real, because generally smaller bodies are associated with women. So men who have smaller bodies, who are, you know, who are, you know, slightly, you know, smaller in size, they mean, even if you take literature, a few novels, I cannot recollect which novel exactly, but then he had a frame of a woman. So there are such descriptions about people who have a thin frame, they're described to, in this sense, like, as if this is lesser, as if this is a deficiency.

So I've seen that representation. And you often find this conversation among people, like people our age, or teenagers, okay, okay, my frame is lesser, I have feminine features, you have masculine features. So I think this has to do with the culture, certainly.

Maybe that's how we were brought up, right? I don't know if it is upbringing or if it has something to do with media, like, just like how I said, I mean, I told you about the novel, right? Where a thin frame, generally, okay, he had a wiry frame. He had feminine features, things like that. So when you read something, you come across something like that in a young age, or when you've been reading such, when you're consuming such content, consistently, from a younger age to an old age, you have started believing in that, okay, oh, I have a smaller frame.

So okay, this feature is feminine, things like that. And, and when we talk about movies, we generally find, okay, this shirt, ripping off shirts to show your abs and body, big bodies and things like that. So I think, you know, offhand manner, they try to emphasize on the fact that, having a bigger body or having big muscles is masculine.

I think they, yes, yes, yes, I agree. Just, you know, I was not only talking about the bringing up, okay. Yeah, this is an amalgamation of everything, I guess.

Okay. Yeah, it could be a visual media. Okay.

It could be a written media. Okay. Of course, literature plays a part.

Okay. Reading plays a part. Okay.

So everything. So I think even my mom, you know, used to say, you should, you are, you are a man. Okay, you should, you should just know, like, she shows this actions.

Okay, you're a man, you should be like a lion. So always, you know, often, you know, males are related compared to lion, male lion. Okay, so so when she says she, it was not only just the, you know, she was not only uttering the sentence, she was also giving me the cues.

Okay, she was saying like this. So it also has something to do with the body, not just because I was biologically born male. Okay.

I should also, you know, you know, project that through my body. Okay, right. That's how it works.

I mean, that's how the norms works in India. Yeah. I have been told things like that, I guess.

I mean, regards to masculinity and femininity, but regarding body image, I think I've

often been told to work on reducing weight, you need to exercise, try to burn calories, you need to, there's no specific reason for that. But then I think, you know, a healthy body is a healthy mind, things like that. So when you when you eat in limited quantities, apparently your concentration level, your ability to your energy levels all improve, according to them, you'll be able to study better.

Generally, being fat is associated to laziness, or a lack of interest in things and stuff like that. So yeah, that is there. So have you ever felt judged or stigmatized based on your physical appearance, particularly concerning your masculinity? You can leave the part of you can leave the part of you know, particularly concerning masculinity, but have you ever felt stigmatized, judged based on your physical appearance? Or anyone teasing you? No one has really done that.

That has been like masculinity. But being fat, of course, like that has happened. As a child, I was extremely fat, obese sorts.

So yeah, there has been mockery, name calling, things like that, tapping on your butt and things like that. Can you please elaborate on some situation where you like really felt judged or you know, like you felt hurt? I haven't felt hurt about that. Because as a child, the idea is to immediately retort to them.

When they call you something, you'll call them something else. You do the same action or do that. So you know, when they will make you feel like okay, this is you're something, you're not normal.

When you're fat, people around you will make you feel like you're not normal. You're not according. They'll say okay, gundubaiya, gunda, things like that.

(25:39 - 28:54)

So this will often happen. So yeah, I didn't feel particularly offended about that. But I felt like okay, I need to work on this is something that needs work.

I need to reduce weight and things like that. Yeah. So do you think there is enough awareness and support available for men who struggle with body image issues in our society? Support as in like, support system, support groups or something like that? Not just that, anything like whenever you know, a woman, you know, talks about her issues,

body image issues, there are people who always know constantly support her or just, you know, say otherwise.

Okay, just to give her a pat. Okay. Because women talk about that.

But I don't think men talk about that. Okay, because they all have this inside. Okay, they have, they have this constant struggle with their body.

And they just try to improve or if they don't improve, they just have this, you know, what do you call maybe a yes, something that is hurting. Okay. They have this inside, but they cannot improvise the same thing.

Okay, and they cannot talk about it. Okay, they just, you know, often being teased, but not retaliate, not to no work on that either. Okay.

So do you think so when I say support, it doesn't have to be groups, it can be anything. It can be just people, okay, or even you know, for example, any policies, because we have all these government advertisements, right? Okay, about all this, you know, child, I mean, pregnancy and stuffs. Okay, so everything we have awareness from the government.

Okay, even especially when it comes to women. Okay, we have awareness, we have awareness about diseases from the government. Okay, because I think male body image is also an issue.

Okay, it's a prevalent issue. Okay, I don't think there is enough support for people who are facing such issues. And I think it's also because they have not really come out on platform saying, okay, this is a big issue.

This is something that affects the society at large. I don't think a lot of people have come out like that. These discussions are generally restricted to limited circles.

Okay, how do you grow a beard? Do you apply coconut oil? Things like that. So usually, with regards to, I'm not sure, I'm not somebody to talk about women, but then, you know, when it comes to women's body image issues, you know, one person can make a change like by coming up with a very detailed account of what they face as a child. There's a lot of support for them.

You find such news articles or Instagram statuses and things like that. I don't know if a

lot of men actually do that. Okay, like I've been mocked for not having a beard, not having a mustache.

I think this is a nationwide problem. A lot of people are facing. I haven't really seen such a discourse.

So yeah, I'm not really sure on that topic. So with regards to support, maybe because of lack of awareness or you know, things like that, I don't think so. I don't think there's a support group.

(28:54 - 29:47)

And it's also because a lot of people are not coming out there to express what they feel. Even today on Instagram, I saw a reel where one oversized woman dancing to a famous song. Okay, and she was getting a lot of support, you know, comments, go girl, like I have seen this morning.

Okay, so you are breaking all the stereotypes, kudos to you. That's what all these comments said. Okay.

But just not long time ago, few days ago, I saw oversized man, okay, doing a song and posting it on Instagram. Okay, but he was receiving all negative comments. Okay, there was no support for him.

Okay, it was actually different. Okay, when it when it comes to women, yes, there are very strong opinions on women. But the days are changing.

(29:47 - 30:06)

And people are supporting for breaking stereotypes for coming out, putting them on the platform. Yeah, but it's not as same as for the men, okay, who put themselves okay, who do not care about how their body may come to the public and you know, project themselves. Okay, so yes.

(30:07 - 30:58)

So there are no enough supports. Support. Yeah.

Okay. So how do you think traditional notions of masculinity affect men's mental health, particularly about body image concerns, traditional notions of masculinity. So we're talking about mass traditional notions of masculinity affecting the body.

Certainly has an impact. Just like how you were saying earlier, about a man is compared to a lion, things like that. I think it's a traditional thing, where it is passed on from one generation.

Your mom probably saw her siblings being praised like that or other men in the family being associated with masculinity, their masculinity being associated with the king of the jungle, the lion, these terms. So I think it is passed on from one generation to another. So certainly tradition has an impact on that.

(31:00 - 33:35)

So what do you believe are the most effective strategies for promoting body positivity and challenging harmful stereotypes about male body image? Most effective strategies. I'm not really sure about strategies. But if body positivity is about mental health, then I'm for it.

But then if it is about normalizing unhealthy behaviors, I'm not for it. So if you have existing conditions like depression, or PCOD, or things like that, if you find a way to tackle that, it is fine. I mean, if you've tried to control your binge eating and all that, that's fine.

But then, if such behavior is encouraged, if that is normalized, like binge eating is normal, I am born this way. So it is okay to when people start normalizing that with body positivity, then I find that problematic. So I think the strategy is to find solutions to change what is unhealthy.

And binge eating and all that I'm not simply saying because everybody is saying, scientifically, it has been proved that it is unhealthy for your mind and for your body, your physical health, your mental health, all that. So yeah, that would be my strategies as such. So from your perspective, what policy changes or societal shifts could help address the gaps in support for men dealing with body image issues, policy changes or societal shifts? Anything that you could just think of or you could suggest? What can be done? I think in colleges and universities, I think it would be good to have a support group discussion of sorts.

We have seen many Hollywood films where alcoholics go to a place where they gather

and talk about their problems, like how they're controlling, and so many years sober and things like that. So we will not be able to do that societally, but at least in universities or colleges, I think we can put some initiative. It doesn't have to be for men alone.

It can be general support group discussion. I don't see that happening anywhere at all for that matter. So I think educational institutions can be the first place to start such initiatives, where you talk about your problems.

It could be anything, mental problems, things that are bothering you, mathematics, educational pressure, weight gain. How do we tackle that? So people have different issues. So just because somebody is physically fit and they fit according to the norms of the society, doesn't mean they have a fantastic mental health.

(33:35 - 35:20)

They might be undergoing something that is serious. So I think it benefits everybody, such initiative can be done. Yeah, so they could start this policy in education institutions, right? Because we have so much extracurricular activities, we have a drama club, we have an arts club.

So why not in a men's club, just not to just gossip, but a support group to talk about anything, to share where men feel comfortable. Yeah, it doesn't have to be a collegewide thing, it can be a classroom thing on a Saturday. And since your topic is about male body image, yeah, we can talk about men.

But I think irrespective of gender, you can make a forum of sorts in the classroom and share your things, education, health, future, jobs, anything. You know, the reason why we felt extremely anxious during the pandemic about our futures was because of the lack of support. We didn't get to meet everybody like we used to in college.

So when we had college regularly, it didn't really bother us, we didn't really think about what's next, because there's a flow to what you're doing. You know, there's a class every day, you have five years of class, you have a certain fixed work period and all that. But during the pandemic, all that was lost.

You know, it's, yeah, so I think that really helps to be in the flow of things. Yes. Okay.
