# Supplementary material for: “An interpretative phenomenological analysis of male body image through the lived experiences of men in India”
Source: BMC Psychol. 2025 Jul 1;13:714. doi: 10.1186/s40359-025-02963-y (PMC12219639; doi:10.1186/s40359-025-02963-y)
Supplement: Supplementary file 2 — Supplementary Material 2. [file 40359_2025_2963_MOESM2_ESM.pdf]

(0:02 - 5:43)

Okay, so this study is called Interpretative Phenomenological Analysis of Male Body Image through the Lived Experiences of Men in India. Okay, bridging policy and practice for gender equality and sexual diversity, that is the aim of this paper. Okay, so you understand this study, right? Okay, so the main objective is mostly, the studies are about girls, women, body.

Okay, so despite growing awareness of body image, this interview primarily focuses on men. Okay, so because men are mostly sidelined. Okay, so this is a research gap in the research which we are going to fill the gap with the current knowledge.

Okay, fine. So this research draws IP, Interpretative Phenomenological Analysis. It is a qualitative study.

So we are going to analyze, you know, using this methodology of the experiences of men. Okay, so basically we have few concepts which will be embedded in the questions.

Okay, you do not need to reveal your names.

Okay, this is highly confidential. If I am going to quote, your names will be changed.

Okay, just be quoted.

Okay, fine. So at any point you can withdraw from the interview. You can pass.

Okay, so just feel free, just do not be alerted or alarmed that you are being recorded. It is just there. Okay, fine.

So I have 10 questions. Okay, you can say whatever you want. Sure.

First question, how do societal expectations of masculinity influence your perception of your body? So in my opinion, society really expects the masculine figure to be like just by body image, they expect it to be more brutish, larger, muscular kind. Right. And I won't lie, I'm kind of aiming for a figure like that myself.

I mean, I have to start somewhere. And maybe I don't know how much I'm influenced by society. But I feel myself better when I think of myself in that kind of a figure.

Yeah. Why is that? Why is that? It's because for one, the culture nowadays is a lot more, what do you call, gym focused. So often I open through social media and I scroll and I see just like reels on gym related content.

And that influences what I want to look like, like a lot. So can you describe any subjective experiences where you feel pressure to conform to certain physical ideals associated with being a man? Your experiences, own experiences, personal experiences. No, non-mental ones, just physical ones.

Anything, anything, but it has to do with body image, anything. It is your experience. That should be relevant to the physical.

Maybe you have certain physical ideals, right? Right, right, right. Okay. So it could be mental or physical thing.

Okay. Like a hacker, a bodybuilder, a sportsman. So I'll give you like the most basic example.

I'm in the lift. Okay. And see, guys in this campus are extremely tall.

Okay. Extremely? Tall. Okay.

Right. So I'm in the lift and the moment the average reaches around my height or like slightly taller than me, I tend to, you know, stretch out my back a bit more because in my mind there's this contest going on and I don't know how biologically, you know, dependent this thing is, but I want to look a little taller than the rest. So just simply out of, I don't, I don't exactly understand why.

But when, when I see somebody really tall, I just instinctively have the urge to try and height up there. So if like the elevator crowd is like slightly above my height, just beyond my tiptoes to like match the height level. That's one thing.

Another would be I'm in the gym, right? I tend to avoid looking at the mirrors because then you see a lot of the other people who are working out there and tons of them have like extremely big arms, everything. Right. And so you like look back at your own hands and you look at your own body and you get like a bit of dysmorphia, body dysmorphia. So I tend to stay away from the mirrors. That'll be another case where I feel like I have to, I can't mentally size up to them. And so my body image and everything, I kind of, I kind of stay, either stay away or try to like fake looking like that.

(5:43 - 10:07)

Understood. So have you have any particular ideals that you worship? Yeah. In terms of

physique, it's like, like you see bodybuilders on, you see bodybuilders in general, right? I follow a lot of fitness related channels, science based, some just like, what do you call it? Based on pure strength, feet, everything.

And these people, they look, they look like Greek, Greek marble statues. Gods. Yeah.

And somewhere within, though, you know, maybe it's not attainable by your own genetic physique. You want to try and look like that. Yeah.

So in what ways do you think media representations contribute to shaping body image perception in our society? I think they make a big difference. If tomorrow looking fat was the cool thing. I think I'd drop going muscle like on the spot.

It would be like such a, and it's not like I'm trying to chase a trend. It's like the trend changes me because now my friends would start putting on like crazy amount of fat.

Right.

And body positivity is a cool thing, but that's different from like, I'm giving you an example of like growing obese would be the cool thing. At that point, I might consider growing obese myself, even if there are health complications. And that's simply because when people's perspectives are twisted in that sense, because of social media, then it hardwires everybody almost.

Okay. That was a bomb statement. Yes.

I mean, I don't want to say that, oh, I'm going to conform to my own health beliefs and everything because at the end of the day, it's like you never really know who you are under the influence of social media and peer pressure. Like right now going to the gym is the cool thing. My friends go to the gym.

I am impressed by their physique and that impression I want to implant into my own self. And so now I go to the gym myself. And under that logic, if my peers were also obese and then social media would project obesity as the cool thing.

So you don't have, basically you don't have interest in going to the gym. It's just because of, you know, how it is, it has become the recent trend that you're trying to... Yeah, I'm trying to just, at the start, like naturally it never occurred to me to go to the gym. It's when I see others I get influenced.

Now I want to go to the gym myself. And at some point, I'll start liking the gym enough to make it a part of my own personality. Right now I'm just trying to chase running towards someone else's image.

Yeah. So have you personally experienced body dissatisfaction or concerns related to muscle dysmorphia? Body dysmorphia is like a daily for me now. Like, I go to the gym, I work out, there's something called a pump.

Okay. And like when you're pumped, you're like double your size, almost feels like, and like, I'll be able to pick up really heavy weights. Right.

And then I come back from the gym, right. And the gym has mirrors where you can see yourself when you're pumped. And under that dim lighting, like that fixated lighting where all your grooves look sharper and everything, you look like a bigger man.

And then you go back to your room, you go back to the washroom, you go wash your face and you see in the mirror, this is somebody completely different. Body dysmorphia is at its maximum. Then you like pose and flex.

(10:07 - 13:07)

And then like, it's not nearly as impressive as when you try and chase for the pump and the lighting together. And that's like, it's a really bad form of body dysmorphia because you know how people say you should compare only to yourself. Yeah.

But right now you are comparing to yourself and you're still unsatisfied. And that's, that's one major body dysmorphia I see. So, do you think, how do you think cultural norms in India impact the way men view their bodies compared to other countries? Compared to other countries? Yes.

I just, I like this, this one point here, which is more on like facial hair. I feel like in other countries, they don't really care if you keep the big beard and the thick mustache. In India, if you keep your clean shave, they'll like call, you'll be like, you look like a girl, this and that, right? So that, I feel like it's one big difference.

Otherwise in terms of body, I think like all over the world, the more muscular you are, the bigger you look. In general, it's like a, what do you call, people tend to appreciate it in general more. So that doesn't change whether in India or outside.

So by adapting to this body image for yourself, by copying others, okay, just to, you know, improve yourself in terms of the image, the physical image, okay. What is the end game? I mean, like, what are you doing? Why are you doing it? Just, you know, keep all these things aside. Like we were saying, because this is what a trend, okay.
This is what everyone is doing. This is what my friends are doing. So, I'm following them. So in the future, if they are going to, you know, become fat, if that is the trend, you will follow that too, okay. Right now, you're following these muscular figures, okay. So, yes, because everyone is doing it, okay.
So you're also doing it. Is there anything more to it to impress, you know, you're doing this to impress your friends or what are the target audience of your newly adopted? There are two target audiences. One, my friends.
To be the bigger man in the room, like psychologically, it really like, it enables, it makes you feel more confident. Psychologically? Yeah, like to my own psychology, to my own psyche, I feel like you're more confident when you're the bigger man in the room, right. Maybe height plays a role in that, maybe, or how muscular you are, okay.
(13:09 - 13:40)
But the second target audience is me, myself, because I feel like even though I am chasing a trend, if, and like, I don't think becoming fat will become a trend. I don't think so. But yeah, so if I maintain at this, at the end of the day, it's my own body health, which I'm improving, and this will have more beneficial effects towards my later years in life, 30-40s and all.
(13:40 - 15:30)
Great. So, have you ever felt judged or stigmatized based on your physical appearance, particularly concerning your masculinity? Concerning my, yeah, when I keep long hair, so for a while, I kept a middle part. Some people said it was looking like a girl, right? Then my, my moustache hair doesn't grow like thick, it grows thin and like really segmented. And that also people would say, I'm not really looking man enough, and that I should consider removing it. And like, at the end of the day, whatever, you know, whatever helps you get through the day without hearing these kind of words, I just follow through.

It's not an insecurity, it's just life is better when you don't have these people inputting thoughts into your head.
Yeah. You were saying all the cool boys? All the cool boys keep the clean shave, right? And then all the rugged manly men keep the beard and the moustache and like it's their pride when they don't shave their moustache. And like, especially in Indian culture, the moustache thing I've seen is pretty big.
Like some of my friends don't go out of their way, like, they won't shave their moustache even if it's like making them, making it difficult for them to eat food. And I don't understand maybe some pride, some Indian pride behind it, some family pride behind it, I don't know. Personally, none of my, what do you call, family members have told me anything like that.
(15:32 - 15:56)
But again, then you'll be like, within that community of people who like really tend after their facial hair, they'll call you stuff for like keeping super clean shave and everything. So that's, I would say, definitely a body image related thing. So you're, so you felt judged, stigmatized? Pretty much, yeah.
(15:58 - 16:16)
Okay, anyone, any person, like, comments that you could think of? So apart from this? Apart from this, yeah. Because they already criticized your hair, okay, moustache, beard. I think for an 18-year-old, yeah, there is nothing to criticize.
(16:17 - 18:15)
I follow through with a similar opinion, but I don't understand what the crowd wants. A lot of people have different ideals. A lot of them like fall under one ideal of wanting to look bigger because, in my opinion, you're freshly 18 and you want to look an adult now. You want to, you want to try and fill in that shoe as fast as possible, even though you should like naturally let it take its time. And so people will follow any route to make sure that they're, you know, from facial hair to the muscle and to the way they walk, talk, everything. They like try to be more adult-like.
Right, so do you think there is enough awareness and support available for men who

struggle with body issues in our society? Do you think, is there any enough awareness? Not enough awareness within the Indian subculture. If you go abroad, there's body positivity, there are men's health awareness issues, every second fitness influencer talks about body dysmorphia and like how to feel better about it. You'll see people like being very supportive, aware about it.

And then here, it's like we're building here. I don't see it nearly as much as like when you go through your more Indian side of social media, you won't see that much. But like there is that vested influence where you'll find more of the, what do you call, body positivity, how to work on body dysmorphia if you're working on this and that.

(18:17 - 19:11)

So there is not enough awareness here. I feel like. So how do you think traditional notions of masculinity affect men's mental health, particularly about body image concerns? Well, for one, if you're a man in general, you don't really get the space to talk about your personal issues, mental health issues.

Maybe you'll have like a selective person or two, but they'll be like your family members and they'll give you like a word of advice that may or may not help you at the end of the day, depending on how orthodox they are. Right. And especially when it comes to body, body health, right, body image.

(19:13 - 20:02)

Like my, my parents, they also want like for a while when I was working out like in school, school time, my mother would like pressurize me to study. And like at some point I just dropped going to the gym at all. Right.

And what else can I say? So you were hitting gym when you were in school? Not really gym. I would like to push-ups and pull-ups, like the bare minimum of exercise. And my mother just, she was like, don't do it during this time of your life.

You will, like you can make a body anytime later, this and that. That time I wasn't looking for a body. I was looking to just get back into, what do you call it? Just get back into general shape.

(20:03 - 21:57)

Because I became really skinny then. What do you mean by general shape? General shape as in a previous version of myself, wherein I was more athletically able. Okay.
I, later on I became super skinny towards my 11th, 10th, 11th. And I was like, like all boon, boon and boon. Okay.
I didn't, I didn't feel good when I wasn't able to do things that I was able to do when I was like maybe a year or so younger. But like my mom said, no. And so this, this family stigma, then the whole study stigma, everything kind of clamped onto me wanting to change my body image, just not even body image, like just my health in general.
So I feel like, and when, especially when it comes to mental health, right. If somebody is suffering from body dysmorphia, there is like in India specifically, there's almost nobody who can like, nobody as a guy wants to approach somebody who can help them.
Because you feel like it makes you weaker.
You feel like, especially because you're working out now, that's like you wanted to do this, right. How are you in a stage where you're feeling bad? So nobody will approach in general. So you were in all shapes, you were in athletic shape, you were in a skinny shape, and now you're in your gym shape.
Yeah. Okay. So have you, you know, getting this constant criticism for all these different shapes of your body transitions? Like when you were athletic, you would have been, you would have received some criticism.
(21:57 - 24:45)
When you were skinny? When I was like, when I was more athletic than anything, that time, nobody like criticized me as such. I received like least amount of criticism I received then, right. Then when I was skinny, it was during the pandemic time.
So nobody really got to see my physical self. It was more about self-reflection. My parents were completely fine with however I look.
And my sister, being my sister, was not involved with this. So that I would say, if I was like going to school, if I was out there more publicly, I feel like people would have said about me getting skinnier and everything. Most criticism I get is now.
Because I'm in the stage of working out where I have to eat more calories and I have to

maintain a certain amount of weight over my normal weight so that later on I can translate this into muscle. But that means I have gotten slightly overweight. Not really, but like I have like a proper belly and every time I walk around without a shirt, my floor mates, roommates, they all tell me that I've gotten fatter.

And this does play a little into my body dysmorphia. So what do you believe are the most effective strategies for promoting body positivity and challenging harmful stereotypes about male bodies? I think the most effective strategy is to just mind your business for all. Let people enjoy, let people live.

In our society, we are like, we are too open about, like we don't, we're open-minded but a little too open-minded and we're too open. And this causes us to like freely communicate these thoughts as positive or like as an insult and sometimes we'll say but like as a joke but it's not really a joke. And then these comments will get passed around and then even though you're not supposed to let it make you feel bad because it's a joke, you feel bad about it because you just, you let it mess with you later on.

(24:47 - 26:20)

Especially with like things like your weight, the ideal muscular physique demands that you have a waist which is extremely tiny. But like for that you have to lose a significant amount of weight. You have to have abs, you have to lose a significant amount of weight to maintain that.

And people don't really like it when they themselves are in a phase where they want to build muscle but instead of muscle they just seem flabby. One effective method is to like somehow teach people to like be more accepting, just like if somebody looks a certain kind of way, they shouldn't like poke at someone. Right.

Then another would be what the western media does which is that they promote body positivity like very heavily. You'll find it within the, like all kinds of media you'll find it within. It's much rarer here but there they'll have like rallies, they'll have any kind of film or anything, they like, they'll subvert with those kind of themes.

You don't see it much here. Here we're still more stereotypical about things. So one thing is following that approach.

(26:21 - 27:33)

Third would be within your peers, if you ever feel like there's something about you that you yourself want to work on, right, like something that from within you're having trouble with like your own image, talk to your peers about it. Because most of them will like encourage you to try either staying the same way or they'll like join you in an effort of trying to better yourself. Right.

That's one thing. That's kind of about it. So in the previous question, you were emphasizing one word consistently.

You were saying have to, have to. I have to, you know, intake calories. I have to, you know, develop, maintain muscles.

I have to, have to, have to. Why have to? Because I'm not there yet and no, it's more like I started going to the gym and there's a process to reaching that figure. And you have to like, have to, again, there's so much you have to do.

(27:34 - 28:44)

And it's a tedious process, but I feel like in some way, maybe like going to the gym or whatever, it's teaching me discipline. Because the process planning, then working and implementing on it, being consistent, all requires discipline. If by working on my body image, I'm able to better myself in more ways than one, then I don't mind going through the entire process, which I'm at right now.

I'm not even like 20% of the way through. Okay. So, from your perspective, what policy changes or societal shifts could help address the gaps in support for men dealing with body image issues? Could you repeat? From your perspective, what policy changes or societal shifts could help address the gaps in support for men dealing with the body image issues? What policy? Policy changes? Yeah, policy changes.

(28:44 - 29:02)

Like a government policy? Yes. Government policy? I don't know about government policy. It doesn't have to be government policy.

Any policy. Just in general? Yeah, in general. Any policy changes.

(29:02 - 30:29)

Okay. I know for a fact, societal change is something that will be done about that. So, for one, to raise awareness about male image, right? Yeah, in general, you'll see all these kinds of advertisements, right? Wherein, you'll have celebrities.

Just take any, like, take a Denver ad, like the perfume, right? You'll see Shah Rukh Khan in there with the body, with the way he looks, that specific body image. And it's like less than the perfume. They're trying to sell the fact that if you look this way, then the perfume will complement you to be even better.

And in my opinion, they should have a policy wherein, not like necessarily about Shah Rukh Khan, but like, more average people should be, like your general consensus, all sorts of people should be in that mode of media, right? Where people in, how do you say, people in advertisements should not be promoting negative body image. Maybe they should have a policy. Maybe a government policy.

(30:30 - 30:50)

Maybe. Okay. Because, you know, it also has to do with mental peace and, you know, your physicality, right? Okay.

So, if the government is concerned about many things, they should also be concerned about this body image because it affects you psychologically, physically. So, yes.

Understood.

(30:53 - 30:57)

Fine. What's your age? Just the last question. 18.

(30:57 - 31:08)

You identify yourself as? Male. Cisgender male? Cis, cis male. Male.

Sexual orientation? Straight. Okay. We can call it heterosexual.

(31:09 - 31:11)

Yes. Heterosexual cis male. Yes.

(31:12 - 31:43)

Yeah. One more point I want to add is that lots of guys wear oversized shirts and they wear it because at some level they don't like the way they look because they don't feel they follow the gold standard, right? And the worst part is you'll see this with guys who

go to the gym as well. At some point of working out, you develop a body dysmorphia that's so bad.

(31:43 - 32:26)

You start wearing hoodies and you start wearing oversized shirts while going to the gym, even if you might be the biggest guy in the gym, right? And a lot of obese people, they also wear extremely oversized shirts so that I feel like they're trying to compensate when there's not much really wrong. Yeah. So that's one thing that I feel like is really, what do you call it? It's like a mental impact that leaves like a choice in their fashion.

(32:28 - 32:44)

The fact that they can't really style much beyond that, beyond those oversized shirts, beyond those hoodies because of the societal pressure that's been put on them about their own body image. I agree.
