# Supplementary material for: “An interpretative phenomenological analysis of male body image through the lived experiences of men in India”
Source: BMC Psychol. 2025 Jul 1;13:714. doi: 10.1186/s40359-025-02963-y (PMC12219639; doi:10.1186/s40359-025-02963-y)
Supplement: Supplementary file 3 — Supplementary Material 3. [file 40359_2025_2963_MOESM3_ESM.pdf]

(0:00 - 0:17)

Shall we start? You don't have to be very cautious about being recorded. This is just there. This is going to be there.

Just you can act, you can talk informally. There's no need for being formal. So I'll just tell you the name of the study.

(0:18 - 1:09)

The study is called an interpretative phenomenological analysis of male body image through the lived experiences of men. So it's basically about how you know the main objective of the conference is to bridge policy and practice for gender equality and sexuality. Policy? Yeah, just you know like policy and practice.

So the ultimate conclusion. So what could be done to create an awareness from the government side, from policy. So it ultimately leads to that.

This is one of the focuses. That's it. So the main focus is it's about male body image.

And we have a method called interpretative phenomenological analysis. That means you are just going to narrate your experiences as a first person. So we are just going to interpret.

(1:10 - 1:23)

I'm not going to just describe it. I'm going to interpret whatever you are saying. So basically this is the study.

It is a methodology. You know methods and methodology. I think you know thematic analysis and everything.

(1:24 - 1:34)

So we will also have thematic analysis here. Like from interpreting your experiences, we will just segregate into themes and interpret it further. That's it.

(1:35 - 2:25)

This is the methodology. Since it is on the title, I'm just explaining it to you. That's it.

This is the basic. And so with all these questions, we are just going to address a few intersections like masculinity, the societal expectations and body image perceptions and muscle dysmorphia and few concepts. That's it.

We have only 10 questions. So the basic research gap is it is always about women and their bodies, their perspectives. So there is no substantial existing literature on male perspectives.

So this study is about that. Using this methodology. This is your whole study.

(2:28 - 2:37)

What's your age? Right now 19, but will turn to 20 this year. Okay, fine. So do you identify yourself as? Male.

(2:40 - 3:35)

Then cisgender male. What is your sexual orientation, if I may ask? Heterosexual. Okay. You think? Straight. Okay, fine. Let's start.

This is just for the understanding. Since you know the title also talks sexual diversity. Okay, so I just needed to know.

Okay, first question. How do societal expectations of masculinity influence your perception of your body? We can just come closer. Okay.

Yeah. For me, sometimes it does happen. The thought does creep in.

I do go to the gym. Like, yeah, here I go to regularly. At home, I used to go to regularly.

(3:37 - 4:45)

And yes, in the Corona time, the Corona period, after doing the 10 standard studies, I, you know, was at home and put on a lot of weight. I was still 110, 115 kgs. So, I mean, it was Corona time.

So, I wasn't much out and about in the, you know, social places. But still, you know, I felt myself that it, you know, it was like a guilty art. Because the social norms have made an image of like a healthy male body.

So, yeah, and also my father, you know, keep kept nagging at me and this is not, you know, this is not good. And he gave me an ultimatum after the 10th boards that you have to, you know, do reducing, you have to reduce your weight. But although we went about it in a, you know, proper fashion, we took a nutritionist and, you know, we had a proper diet.

And for the next nine months, actually dropped down to 94 kgs. Yeah, with the help of

proper nutritionists. Yeah.

(4:47 - 5:23)

Understood. I mean, my father is also a man of science. He's also an atheist.

So, you know, he just follows science. And, you know, the logical choice was having a nutritionist. And he also understands everything.

He likes to understand everything, how it works. So, was he like critical about it or just wanted to be supported? Critical? No, my father is, I personally don't think he's ever critical. Even when it comes to marks, he just does not care, but he just expects me and that itself motivates me to do good.

(5:23 - 5:51)

Okay. So, that's the thing. He never asks about the marks.

I just tell him. My mother, on the other hand, she asks for the marks. But yeah, my father, he just expects something from me and I just, you know, I don't try to disappoint him.

And that was the same thing about the weight. I, you know, my father, he's in a healthy shape. He's been going to the gym regularly, even on vacation when he comes to India, he subscribes to the gym and he goes there every day.

(5:52 - 7:08)

So, looking at him also, I felt motivated at that time also. That was the thing. So, can you describe any subjective experiences where you felt pressure to conform to certain physical ideals associated with being a man? Just your personal experiences where you felt, you know, you have this constant pressure to conform to the, you know, the physical ideals, right? It could be a hero, okay? It could be any hero, like Shah Rukh Khan, someone who has this perfect body, six-packs, okay? Or any, you know, like influencer or just, you know... Not only influencer, I was in a hostel most of the time.

So, the people around me was the main sort of pressure, if you could say. So, in the 10th or 12th, sorry, 11th and 12th, there's a, you know, a class called, a course called PE, Physical Education. But the thing is how I, you know, reacted to it, I didn't take that course.

So, I just avoided that. And I was doing my own thing and I had my weight at constant at 94 to 96. It didn't go above 100.

(7:08 - 8:31)

And I personally don't think the social conventions, you know, impact me much. But always there is that subconscious nagging in the back of my head that, yeah, I have to reduce my weight. But then at the end, you know, the laziness takes, laziness becomes, you know, overcomes that feeling of social pressure for me, especially.

So, I thought, I think right now, I'm at a good place. But still, I need to reduce. I know that I need to reduce my weight.

Right now, I'm at 96 to 98. It keeps fluctuating. But right now, it's staying there.

So, it's in my future plan to take it to, you know, in the 80s somewhere. But that's, you know, I also have to take, you know, my surroundings in consideration. Because, you know, hostel or, you know, it's not possible.

Because the food there, you can't control it. Because I have reduced my weight by, you know, proper control of my diet. So, like, you have to exactly match the number of calories, the number of carbohydrates, the number of proteins, what the nutritionist gives.

Or whatever you set it for yourself. But that's not possible in the hostel. So, do you have any worshipping ideas here? Like, not just here, but in general, right now, like you have someone in mind.

(8:31 - 9:40)

You just want to adopt their lifestyle and maybe their body figures. It's some idea. Because, you know, if you want to become an actor, you must be having an ideal, right? If you want to become a scientist, if you want to become a good engineer, architect, there should be some idea, the thing is, one interesting, you know, field of job, one interesting field I'm interested in is chefs.

They don't have very healthy bodies. Not always, some do have, but yeah. And, no, my most, you know, the most inspirational is my father, sir.

He regularly goes to the gym. So, he's your ideal? Yeah, he is. And he also motivates me.

So, that's closer than ideal. What do you mean by healthy body? Healthy body, yeah. A healthy body is something that you just, you know, get.

It's not like, it's, you know, it's subjective to every person. It's not same for every person. But it's something that you can maintain over a long time by, you know, eating healthy.

(9:40 - 10:11)

Eating healthy as in, you know, there are some scientific, yeah, some, you know, the amount of calories you need, the amount of protein you need, that if you follow every day, eventually you will come to a point of stagnation where your weight is not going up, does not go down. You know, you are living healthy and you don't get body aches or you don't have headaches or you have a continuous proper sleep cycle. That's, you know, that's the idea of a healthy body.

(10:11 - 10:40)

And so, because when you were talking about the chefs, right? Like, when you see them, they don't have... Like, they are very fluffy. Okay, they are fluffy, but how do you know they are not, like, properly managing their diet chart? Maybe if they are, maybe they are eating healthy, but maybe, you know, maybe for females, they have, they might have PCODs, which is, which, you know, eventually will be the consequence of them becoming obese. Yeah, the thing is, I don't know that.

(10:41 - 11:00)

So, the thing is... So, when we just... Yeah, just we see, whatever we see, we think on that. So, yeah, there are chefs which are healthy and in the fit. That's also a part, but majority are like, which we have our attention towards, they have fluffy bodies, yeah.

(11:00 - 16:02)

Okay, understood. So, in what ways do you think media representations contribute to shaping male body image perceptions in our society? Just the role of media, you know, how it affects your perceptions in our society, in the cultural context right now? Yeah, media is a tough thing, because right now, it's more, you know, more than, more easier than ever to, you know, get on to social media, and especially Instagram and Twitter, they idealize the body, but that, that also depends on, you know, your outlook yourself.

So, if you, you know, spend too much time on certain types of social consumption, like especially in the gym or the deals which portray, you know, healthy bodies and all, there are some things that, you know, they make you want to have, make you want to have a good, healthy impression of yourself, but I think that is also, you know, a plus point.

So, that would make you want to have a good body. If you have any, you know, diseases or, you know, any problems, which don't, that does not apply to you, but if you have a normal functioning life, functioning body, then, you know, having a goal to reduce your weight or, you know, make a healthy body, that's always a plus point. That's always a good thing, even if that is coming from social media.

Okay. Cool. So, have you personally experienced body dissatisfaction or concerns related to muscle dysmorphia? Body dissatisfaction? Yes, every day.

But the thing is, yeah, my muscles are fine. Muscle dysmorphia is not a big issue for me. But the body image, yeah, I look at it every day.

I feel it. I feel my tummy. The thing is, right now, I know that I am eating healthy and, you know, that's the same thing that my father has always taught me.

You have to just think about eating healthy. It's not about, you know, how fast you reduce your weight or how quickly you become to fit. Because when I was in my, you know, when I was reducing my weight every day after, you know, after gaining it to 110 kgs, we actually had a target.

So, in fitness, there is a target you need to achieve. It's like only 0.5 kg per week you have to reduce. Because if you even lose your weight too fast, it also affects your health. Adversely, it affects your health. So, the thing is, you have to even go into reducing your health in a healthy way. So, that's why we also, you know, took help of a nutritionist.

Okay. So, body dysmorphia is something that, you know, it's a, you can say it's a condition or it could be a disorder where you feel this constant, always think about your appearance, okay, and you always think that you are too small or maybe not good enough, feel good enough about your body. Yes.

Basically, like you feel so embarrassed or anxious about in the social conditions. Even for teenagers, I think so, you know, the facial, the pimples and all are also part of body

dysmorphia that might lead to insecurities. But for me, I was away from social media at the, you know, for most of the time.
So, I didn't go into Instagram at all and whatever I went into Instagram, it was for very little time. So, yeah, body dysmorphia does play. I've seen people in real life who, you know, keep on, you know, trying to make themselves, you know, beautiful.
They take care of their skin care, you know, properly. But the thing is, it's a natural phenomenon, you know, if you get pimples or if you get this thing and yeah, it, the social media does play an impact on people which they want to, you know, take care of themselves or they want to become like other people, even if it is not achievable, but that is another thing. Okay.
So, just leave social media and everything like just, you know. Societal conventions.
Yeah.
Societal conventions, those are the main thing. People do have an idealized image on how they want themselves to be like clear skin or fair skin and especially, you know, the advertisements also target them. They want clear skin or, you know, soft hair or those things and that might not be achievable for every single person and that creates a, you know, bad thought or bad image in people's mind.
(16:02 - 18:27)
Hmm. Maybe the sense of detachment. Yes.
A sense of insecurity of themselves and that might lead to, you know, lack of confidence or, yeah, lack of confidence of themselves. Yes. Okay.
So, how do you think cultural norms in India impact the way men view their bodies compared to other cultures? It could be, you know, like from other states or other countries because there is a difference between, you know, how they view their own body, right? So, if you are in South India, you have to be like, you have to have this build.
Okay. You have to, like, dress this way.
You have to have this body image. Yes. When you go to North India, it's different.
When you go to abroad, it's different. So, how... Yeah, culture does play an importance, especially when you are getting married because everyone keeps you reduced weight,

look good, especially during the marriage time. That does, you know, play a big role for this thing.

What was the question? Yeah. How do you think cultural norms in India impact the way men view their bodies compared to other cultures? So, how the cultures impact your perspectives about you viewing your own body? So, the Indian culture with the Indian dances and the Indian costumes, you know, they are very different Indian costumes all across India. So, yes, and the costumes... When you say cultural norms, it could be anything, even the food also, right? Yeah.

That also comes into the food culture. So, we have many cultures. When you say culture, it's not just an art and music.

Even the food itself is a culture. So, anything, even the way we consume food, because all the food from North India and South India, they are different. Yes.

Some food is heavier than other. The quantity of food which they get, you know, served during this thing at all at home. But at the end, even though, you know, I think it boils down to how people think of themselves and how ingrained is culture in them.

(18:28 - 19:10)

So, like, if they are, you know, very cultural, they will, you know, wear the clothes, they will try to, you know, look at the idealized figure. And that does play a role. I'm not sure about how Western culture views male bodies.

Right now, Western culture is, you know, very diversified. They, you know, they are inclusive of all of the... They are inclusive of everything, if I can say. But in India, still, you know, it's still a developing country and people do stick to culture and that does play a very big role.

(19:10 - 20:10)

People. So, have you ever felt judged or stigmatized based on your physical appearance, particularly concerning your masculinity? No, not my masculinity, but, yeah, physical appearance, yes. I am fat, all obese, however you put it.

But, yes, it all comes back to, you know, how I was brought up. My father has always ingrained confidence in me. And, yes, I know that I'm trying to do the right thing by

becoming healthy.

So, that just makes me not care about other people's opinions. So, that's one thing. But, yeah, I have seen other people, you know, get very tensed about, you know, how other people view them.

So, yeah, that's the thing. So, like, you never felt like... You don't care about any... I don't care. I have felt it.

(20:10 - 21:15)

Okay. So, like, how it happens? How the stigma or judgment happens? Yes, like, just when you're on a video call with your relatives or something, they say you've put on weight or you're fat. It just puts a thought in the back of my mind that I have to again stop eating or again have to go on a diet, which is obviously an uncomfortable thing because I am a very big foodie.

And, yeah, that's the main thing. Why is that? How? Because, you know, like, even all the other interviews, I have just noticed this. They have consistently been using the word, how.

I have to. How do I have to? Like, that's the norm. And that comes back to the societal pressure that is being put on them.

So, they think they have to. But then at the end, it just comes down to the individual's personality or individual's mind or whatever they think of themselves. Because if that force is, you know, very great, they will obviously try to lose weight or, you know, go towards the idealized body.

(21:16 - 21:22)

So, that's the how condition. How condition. I have never thought how would be the condition.

(21:24 - 21:28)

How to. Even, yeah, I have to reduce weight. I have to.

(21:28 - 21:32)

I think maybe we could rephrase it. Maybe. I want to reduce weight.

(21:32 - 21:57)

That's very, that's very. All you can say, it's best if I reduce weight. Yeah.

Because if it's best to, you know, follow a healthy diet. Yes. It's not like that.

It's like I have to look good to impress. I'm the only one person with the thing. I want to reduce my weight.

That's my father. I'm the only one person. So, that's the thing.

(21:58 - 22:33)

Okay. So, recently he tried to, you know, just he also, you know, watches like a good model. He idealizes the body.

Recently, he wanted to, you know, get body fat below 10 percent. But the thing is, he went on a nutritionist plan for that. And the first step was to increase his weight.

But the thing is, how much ever he ate, he could not increase his weight. So, that went on for three months. He tried eating a lot.

At the end, he just gave up on that. Because being healthy is enough. I don't want to, you know, reduce my body fat even more.

(22:34 - 22:39)

That's fine. I'm healthy right now. So, right now, he's just maintaining his weight.

(22:40 - 22:46)

How old is he? Right now, 48 or something. Yeah. Okay.

Young. Almost 50. Young still.

(22:47 - 33:00)

I was really young still. Do you consider Shah Rukh Khan and Salman Khan as young? They're still acting. They're still, like, acting with all these 20-25 years old actresses.

I don't see anyone calling them out as old people. But my father has a large body. So what? It's just a matter of weight.

If you have too much money, you can cover it. Yes. So, do you think there is enough awareness and support available for men who struggle with body image issues in our society? The awareness could be something, like, somewhere.

It could be from somewhere. Anything. Do you think that there is? Like, no.

Personally, I... In certain fields, yes, there is. Like, some newspapers have a certain

section of fitness or, you know, healthy diet, healthy eating. Newspapers have.

But the thing is, people don't really, especially the up-and-coming teens or the, you know, children, they don't really read newspapers right now. They just go to social media, which social media has the, you know, all the algorithms behind it that it just shows the people what they want to see. It does not show everything behind.

So that does, you know, impact. So, question again? Yeah. How do you think, sorry, do you think there is enough awareness and support? Awareness and support.

Yes. At the end, it all depends on where a particular, you know, individual is residing or who is around them or how good is their support cycle or how good is their support cycle. And for me, I had my father, but everyone does not have a very fit, conscious or a very healthy-minded parents or healthy-minded friends or peers or everyone around them.

And yes, I have seen some places where there is awareness in social media also, but majority, I still don't think that there is much awareness about, you know, the male body fitness or the muscle dysmorphia or all the conditions that cause, you know, other types of bodies. So let's say if you watch some series of movies, which deals with the mental conditions, suicidal thoughts, you will find these links every time. If you know someone or if you are that someone, please feel free to mail us or get the support from this.

So just like that, you know, if you, I still remember, you know, watching The Crown. Have you seen The Crown? Okay. So The Crown or anything that is like, because you know, one of the scenes which, you know, the princess Diana, she has an eating disorder.

It was called something. I forgot the condition name. Okay.

So she has this eating condition. So when she started eating, she just needs a lot, lot.

She just stuffs everything inside.

You know what she does? She just throws up. Intense. She tries to get it out.

It's not like she's just not, you know, digestive. Yeah, yeah, yeah. Again.

Yes. Just not, I don't want to put on weight. So whatever I consume, I just, you know, like throw it out.

Okay. So whenever that scene, which deals with her eating disorder is, there is this, if

you have eating disorder or if you know someone who's going through this, please feel free to contact. So something like that.

Have you ever come across something like that? Any awareness of that sort? Yeah, there are, yeah, I've come across that in movies. Which one? You're talking about body image. There is nothing, right? I mean, yeah, I haven't seen much of them.

Cool. So how do you think traditional notions of masculinity affect men's mental health, particularly about body image concerns? Traditional notions of masculinity, like how culturally acceptable, how a man should be, the heteronormative. Like in this century, we are going towards, you know, all inclusive activity with LGBTQ things in the Western culture.

Right now it is picking up, but still in the Indian culture, the traditionality still maintains the idea of two-gendered ecosystem, whether it's only female or male. And that is really detrimental to the upcoming of the society. It does not allow the development to pass very much because that's natural and you cannot stop it.

But then the people feel that due to traditional stigmas or traditional forces or cultural forces that they have to adhere to this thing, even by putting themselves at discomfort or not liking it. And that does create, you know, sometimes that does create suicidal thoughts or maybe, you know, extreme cases. In extreme cases, it's suicidal thoughts or they just, you know, hide all the feelings in the back of their mind.

That's just, that's the main thing that traditionality, you know, has an impact on the male image. And that also carries over to the female images, the traditionality in India especially. So, what do you believe are the most effective strategies for promoting body positivity and challenging harmful stereotypes about male body? Understand the question.

Just, I just wanted to think on what do you believe are the most effective strategies for promoting body positivity? Most effective strategies would, in my opinion, will be like target, you know, mass sports like cricket. Just put, you know, advertisements on the IPL for, you know, all the inclusive activities. Right now, yeah, they are coming, the LGBTQ, the month, I think so next month is the Pride month.

I'm not sure what they are going to do for it. Last year, I haven't seen much of it or was it there? I'm not sure. For the, you know, all the cricket matches that are this thing, they just advertise them like that is for cancer.

Right now, yesterday, I think so the Gujarat team, someone wore pink shirts for, you know, the cancer awareness. Similarly, I think so, you know, hitting the mass media like using sports or, you know, using, yeah, mainly sports and media users like movies.

These are the two main things that, you know, is like very mass because nowadays even no one, I personally don't see many people reading the newspapers, around me especially.

So, those two are the main ideas. That should go into my conclusion because it makes sense because, you know, I think other people didn't think about that. Because there are 3-4 crore consummating these years in the cricket IPL.

That would give a very wide range and especially the people who watch cricket are mostly men. Sports in general. Yeah, sports in general are mostly men.

But yeah, women also watch. But yeah, and those men also idealize the cricketers themselves, right? So, that the cricketers themselves are showing a message that it's fine or, you know, that it's okay to have disabilities or dysmorphia or these things that would have a very good impact on the images. Yes, exactly.

I second that. So, from your perspective, I think you already answered this question kind of. It is the same, okay.

From your perspective, what policy changes or societal shifts could help address the gaps in support for men dealing with body image issues? So, what are the policies or societal shifts that you think that could address, you know, the gap in the support for men dealing with this body image issues? It comes down to marriage, like homosexual marriages or just being inclusive of the whole LGBTQ community. That's one of the main thing. Other is for men, especially policies.

I mean, it's more of a personal effect, like how people view themselves. I don't think policies would, you know, alter much in that department. So, mostly just advertisement in mass media will be the most useful.

Otherwise, policies won't be much helpful in the personal image of themselves. If they don't have any, you know, body dysmorphia or the muscle dysmorphia or other conditions like that. If you have conditions, then especially the hospitals or the clinics or they have to put up the advertisements or, you know, just to information for everyone that this can happen.

And if you have symptoms like this, they can also approach the healthcare to, you know, get it fixed if it is a fixable thing. So, it's like if it's untreatable. So, like if you have, you know, like STIs, STDs or HIV, like, you know, instead of just stigmatizing it or just, you know, we can, you know, create an awareness, make policies to support, right? So, it's similar to that.

(33:00 - 33:32)

The thing is with the Indian, it's very hard and it will take a long time. It's not very easy because people are very traditional, they are very cultural and especially reaching all the, you know, all everywhere in India, it's very difficult. It would be possible in the big major cities.

Very easily, it's possible in the big major cities. Right now, they are going towards, you know, inclusivity. They are going towards all the advertisements or whatever.

(33:32 - 34:15)

But the thing is the small villages and all, those are the, you know, those should be the target for all the advertisements or whatever information that we have to provide related to this topic. Maybe, I think they should also advertise like how they advertise other socially, you know, like social awareness things, okay? Like whatever the diseases or anything, even women empowerment, they all have this public advertisement, right? Maybe, they could. Yeah, they can, sir.

Yes. That's the main comparison which you could do. Right.

That's all about it. Thank you.
