# Supplementary material for: “An interpretative phenomenological analysis of male body image through the lived experiences of men in India”
Source: BMC Psychol. 2025 Jul 1;13:714. doi: 10.1186/s40359-025-02963-y (PMC12219639; doi:10.1186/s40359-025-02963-y)
Supplement: Supplementary file 4 — Supplementary Material 4. [file 40359_2025_2963_MOESM4_ESM.pdf]

(0:03 - 0:31)

Okay, Mr. J****, do you have any idea of this research? I believe it is about how men feel about their body. And overall, I think it is a fitness-based research. I mean, so trending, body shapes, which is being impacted in terms of fitness business.

(0:32 - 0:39)

Okay, good. That's right. Okay, so I'll just state the name of this research.

(0:40 - 0:59)

Okay, it's an interpretative phenomenological analysis of male body image through the lived experiences of men in India, bridging policy and practice for gender equality and sexual diversity. So the main concept here is a male body image. Okay, in the context of Indian people, especially Indian men.

(0:59 - 1:12)

Okay, so how they perceive basically how they perceive their body. Okay, and all the morphias, dysmorphias. That's what whatever men dealing with, okay, with respect to their bodies.

(1:12 - 1:27)

Okay, because when you look at the research part or any sociological perspective, it's always about women. All the societal issues are about women. Okay, so all the supports and everything regarding women are there.

(1:27 - 1:41)

There is nothing about male body image. There is no topic or topic of discussion about male body image. So that is one of the gaps in the existing literature or in the topic forum, speech forum.

(1:41 - 2:00)

So that's why this research is based on male body image. And do you have ideas about methods and methodology in research? Both qualitative and quantitative research. This is a qualitative research.

(2:00 - 2:13)

Okay, so we have employed an interview. Okay, especially we have adopted the method

called interpretative phenomenological analysis. Let's say like you experience something that is your first interpretation.

(2:14 - 2:23)

Then you're narrating that experience to someone or to me. That is your second interpretation. Then I interpret whatever you have told me.

(2:23 - 2:32)

That is third interpretation. So to make sense of that interpretation is called interpretative phenomenological analysis. So this is the basic idea of this research.

(2:33 - 2:43)

Okay, the pattern of this research. I just want you to understand where we are going. So please feel free to share your opinions, your experiences.

(2:44 - 2:56)

You don't have to be conscious of being recorded. You can be free. I'll just start with the first question.

(2:57 - 3:11)

You can deviate from the questions. Okay, sure. So how do societal expectations of masculinity influence your perception of your body? Societal expectations of masculinity.

(3:11 - 3:36)

How masculinity should be. So the society has some rules and regulations on their thoughts of masculinity, right? So how does it influence your perspective or your perception of your body? Yes, definitely. Men do have some societal expectation.

(3:37 - 3:52)

And which is something we cannot even talk, we cannot even deny. For example, let's say there is a heavy object lying on the ground. And nobody's going to, I mean, I'm not stereotyping or anything.

(3:52 - 4:04)

But it is a basic expectation of a society on a man to lift that object. And move it from the place. So they don't expect a woman coming forward and putting forth themselves to move the object.

(4:05 - 4:25)

So for that, if a man is lean, if a man is not very good with their physique, they are not incapable of moving the object. Or they're not capable of helping the others through physical work. Definitely he is going to be looked down.

(4:26 - 4:49)

And we know it is very common, as we say, the things which we usually talk about is when we call a woman fat, then it is going to be offensive. But it is not much of a knowledge when lean boys or fat boys are being called fat or anything. There are people who stand up for themselves.

(4:49 - 5:10)

But once again, when it comes to health and everything, the perspective definitely changes. The way we encourage and motivate them to stay in shape is different. So when it comes to women, at least they have a voice.

(5:11 - 5:20)

People are voicing out. But men, we are expected. People are not even ready to tell you about encouraging.

(5:21 - 5:40)

You are just expected. So yes, the societal expectation, definitely it is increasing day by day. Because the definition of fitness and the definition of the perfect, Mr. Perfect, the body of Mr. Perfect, the expectations are going up and high.

(5:41 - 6:00)

Though there are some resistance with accepting. But one thing we have to be very conscious about is that it is not about fatphobic or anything. People say that you don't have to be fit or you love your own body.

(6:01 - 6:14)

But definitely, if you love your own body at the cost of your own health, then there is no point in loving your own body. If you love your body, you have to take care of it. This is where the corporate comes in.

(6:14 - 6:24)

They use this space and they manipulate us to get into a particular shape. That is not fitness. That is not health.

(6:25 - 6:44)

So yes, pointing out my major key points. First is the societal expectation is due. They do have certain expectation on men's body and unspoken societal expectations, which are expected from men to be understood by themselves.

(6:45 - 7:21)

So yes, and also the influence of corporate and other companies to make men to feel this is what perfect is. So can you describe any subjective experiences, your personal experiences where you felt pressured to conform to certain physical ideals associated with being a man? Meaning, like we all have this, what do you call it, ideals, right? Personality. So this person, I want to be like this person.

(7:21 - 7:31)

He has a very good physique. He's very fit. So we have this bodybuilder or any actor who has packs, who has built his muscle very beefy.

(7:32 - 8:14)

So I want to be like him. So do you feel any pressure as a personal experience where you had to conform to this perfect ideal of person? Personally, I always wanted to stay healthy. But when I move into the society, what I find, see, if I know I have excess fat in my body and I know there are fats sedimented over my hips region and everything, but I can do 30 sit-ups, which shows that my lower body is strong and I am staying healthy.

(8:15 - 8:31)

So I personally don't want to get into the particular shape. But the usual question asked to me is that, are you going to gym? Yes. Then why do you have a fat tummy? What? I don't know.

(8:31 - 8:38)

Maybe I'm just trying to get in shape. That's it. So these are the expectations put forward on me.

(8:38 - 8:59)

Also, you can see me, right? I have a decent shoulder and everything. The first thing people think out of me is that, so we can trust you in a fight, right? Why? I don't know who you are. The people come and talk to me like this.

(9:00 - 9:15)

They expect me to be strong. They expect me to protect them whenever there is an issue. I'm not saying that I'm kind of a hero or anything, but by looking at my appearance, I'm not even comfortable with people touching me.

(9:15 - 9:28)

I have certain things. Not everybody is comfortable with that. So certain expectations put me into, it is kind of a phobia for me these days.

(9:29 - 9:39)

I cannot even go into the public. If I go to the public, the random uncles or someone just tells me, look how strong he is. No, I'm not strong.

(9:39 - 9:43)

I'm just trying to stay fit. I'm just trying to stay healthy. Look how strong he is.

(9:43 - 9:52)

He can even beat us now. Why should I? So these are the things which I find spoiling my reputation. I don't find it.

(9:52 - 9:57)

Some may find it, they are praising me. Okay, see how strong I am. No, I don't like those kind of things.

(9:57 - 10:16)

I don't like this unwanted attention because I fear the day when someone pushes me down, they are trying to create some kind of bubble reputation on me. So when it breaks down, definitely I don't want it to damage me. I don't want to believe myself.

(10:17 - 10:24)

I don't want to have the head bite or anything. I don't want them to create it. So I'm being very cautious of that.

(10:25 - 10:49)

Also, one more point. I would like to add one more thing. I personally find that when I, with a particular mindset of staying fit, staying healthy, moves into the environment where people describe something as this is healthy, not what you think is healthy.

(10:49 - 11:19)

When I move into the majority of people with the orthodox mindset, not orthodox, with a contradicting mindset of what mine is, I intend to move into it. I have to follow them consciously or subconsciously or somehow. I'm afraid that they might infiltrate into my ideas and it might have certain influences on me.

(11:20 - 11:31)

So I'm not able to recognize it yet. But yeah, definitely I'm going to notice some difference when I'm surrounded by those people. Understood.

(11:31 - 11:35)

You were saying something. Okay. You said, I think, decent shoulder.

(11:35 - 11:56)

Can you describe what decent shoulder is? What decent shoulder? Yes. What a healthy shoulder is that? A shoulder with a good coverage in muscle. So you can see the shape of the shoulder varies from person to person.

(11:56 - 12:01)

It can be round. It can be an oval kind of shape. It can be broad.

(12:01 - 12:27)

The shape of the shoulder doesn't mean there is no ideal, perfect shoulder. But the size, the V shape, the shape V gives with the broad shoulder, comes with a broad shoulder and a lower waistline. So if you have a broader waistline, definitely, which means that you're going to, now or later, you're going to face diabetes.

(12:28 - 12:50)

So you have to take control of your waistline. And by the same way, in case to showcase of your broad shoulder, you definitely have to have a smaller waistline. So this is not mine, but the expected outcomes of muscular shoulder is that the shoulder has to be broad.

(12:50 - 12:59)

And it has to be muscular, not clumsy. Why does it have to be? You said it has to be. It has to be.

(12:59 - 13:11)

Yes. Or else, whenever you're going to lift weight, even I have a shoulder injury. It is because I failed to train my shoulder.

(13:12 - 13:34)

So if my shoulder is muscular and it is broad, I have the strength to cope up with my body. So whenever I find lifting weights, since you have a very good upper body strength, you can lift the weights easily. But when the shoulder is not ready to cope up with the weight, you're definitely going to face some muscle tear or ligament.

(13:38 - 14:01)

So do you have any, can you name or point out your ideals where you just want to adopt their body? Do you have any ideals? Yes. I am always amazed by the strength of these calisthenics. They are very good.

(14:03 - 14:35)

I'm not going to elaborate on the weightlifting or anything, but they are very good with these ground exercises, balancing themselves, carrying their own body weight by their hands. The way they balance in a double bar, let it be in a monkey bar, that is one type of strength. Not the body shape or anything, but I want the strength, I expect the strength to lift my own body in a certain way.

(14:35 - 14:53)

That is my least expectation. In what ways do you think media representations contribute to shaping male body image perceptions in our society? The role of media. Definitely.

(14:54 - 15:42)

Media has a huge role to play. Because all the false expectations, 90% of these, how do I put it in words, the ideal body, the expectations of the society, everything is being embedded in the true media. Let's say for Instagram, I recently happened to watch, a

guy was posting for his feed, and he shows, this is how I look in Instagram, this is how I present myself in the reel, and this is how I usually be in my normal days.

(15:43 - 16:06)

You can see the tone difference. He just holds his breath to showcase his muscles and everything in Instagram reels, through reels. It is believed that by showing off those muscles and everything, it fulfills the purpose of the reel, because it is about how to look muscular and everything.

(16:06 - 16:29)

But that is not the way they usually live. After the seconds of taking those reels, he has this tummy fat and everything, because he is not going to post for the entire day. What we mostly see is that, the body he had while he was posting for the reel.

(16:29 - 17:00)

This is being projected by the media, for the past few years. You can say, for example, for underwear advertisements, you are never going to see a man with a hairy chest or a fat tummy. That body, that projection of male body, nobody is going to have it, because nobody can attain it.

(17:00 - 17:16)

There is one saying, you know why gym is addictive for me? Because you can never reach the pump. When you work out, you can see the muscle pumping out. But after you take rest and everything, the pump goes away.

(17:16 - 17:22)

When you work out the next day, the pump again increases. You can never attain the pump. You can never reach the pump.

(17:22 - 17:32)

If you want to reach the pump, you have to work out more. So in that case, you can never reach the particular, the body which the media portrays. That is not normal.

(17:32 - 17:58)

And I strongly believe that people expect this. So if you go to the gym, you go to the gym, and definitely you are going to get this type of, you know, the body which I, which

the media usually projects, is that the man with a clear definition of his chest, with at least the sixth definition of abs, and broad shoulders and everything. This is not normal.

(17:58 - 18:04)

That is not normal. That is not at all possible. You can flex those muscles only for a minute or two.

(18:04 - 18:11)

That's it. You can even watch the bodybuilding competitions, right? Those muscles can be flexed only for a minute or two. That's it.

(18:12 - 18:21)

I'm not sure if it's a minute or two, or maybe five minutes, I don't know. But that is not normal. And the major role making these things normalized is played by the media.

(18:22 - 18:47)

I say that. Through advertisements. In order to even, I don't know if I can speak about it, but even through pharmaceutical companies, right? How come, even there are questions, how peanut butter enters into the response to your fitness? There is no, because there are other multiple ways to attain protein heights.

(18:48 - 19:01)

But they use, they advertise these chemicals, medicals. See, the ultimate thing is to stay healthy. But the media projects that use steroids, use this, use that.

(19:01 - 19:11)

How can we be healthy by using steroids? Those are for medications. Those are not for building your body. There are certain things.

(19:12 - 19:32)

So let's say, let's say, if you have to, let's say, if you have to attain protein, you have to eat certain grams of chicken to gain minimum grams of protein, 100 grams of chicken, 25 grams of protein. Okay. And the media projects that, the media says that, why do you have to waste your money on chicken? Get some, buy some protein powder.

(19:33 - 19:53)

One scoop of protein, 25 grams of protein. Then your digestive system is going to adapt

to itself, to that, right? It doesn't have to struggle a lot to separate meat and protein and digestive system to take the protein separately. No, it doesn't have to work hard.

(19:53 - 20:10)

All it has to do is consume the protein powder. That's it. So those things can be attained naturally, but the media projects in a certain way that, why do you have to struggle with that? Why do you have to go through all those struggles? You have to cut and clean the chicken.

(20:10 - 20:16)

You have to cook the chicken. No, just take a scoop. This is a minor example.

(20:16 - 20:35)

No, I don't want to get deeply into that. I don't want to make things controversial yet, but this is a minor example. There are so many chemicals and other steroids are being advertised in a certain way, not in the name of steroids, but with a particular brand name, so that it doesn't seem harmful.

(20:36 - 21:18)

Yes, media definitely do have a greater role to play in this. Have you ever personally experienced body dissatisfaction or concerns related to muscle dysmorphia? Muscle dysmorphia is a condition or it is a disorder where you feel, this constant comparison with yourself or with others, where you feel you have a shortage of muscles. Though you are beefy, let's say, sometimes you are beefy or you are somewhat built, you have muscles, but you always think of yourself as short of muscles, that you are not well built.

(21:18 - 21:39)

Even if you go to the gym, you look at others and this constant comparison is there, that you are never enough when compared to others. That has something to do with your mental condition, your mental well-being. When you look at others, when you look at yourself in the mirror, it's never enough.

(21:39 - 21:44)

It's not enough. I'm not enough. I'm not capable.

(21:45 - 21:54)

This is a disorder. We have body dysmorphia. Muscle dysmorphia is one of the dysmorphias in body dysmorphia.

(21:54 - 22:10)

Have you ever personally experienced this body dissatisfaction and muscle dysmorphia? Body dissatisfaction, yes. But muscle dysmorphia, yes. Body dissatisfaction, yes.

(22:10 - 22:26)

But muscle dysmorphia, I wouldn't put it that way. Because I have a very lean wrist. And I always wanted my wrist to be bigger so that I can avoid injury.

(22:26 - 22:39)

Because I had injury on that. The reason for that is that I have a very small wrist, which couldn't cope up with my body weight. So, yes, I always wanted my wrist to be a bit bigger.

(22:40 - 22:49)

And I do work for that regularly. But it is never enough. I wanted it to be bigger, much more bigger.

(22:49 - 23:02)

So, it was never enough. Maybe, yes, that is one of my body dissatisfaction. And muscle dysmorphia, no, I don't think.

(23:02 - 23:08)

So, you don't compare. You don't go to the mirror. You see yourself and you're not satisfied.

(23:09 - 23:13)

That is also muscle dysmorphia. You go to the mirror. You just do this, do that.

(23:13 - 23:26)

I wouldn't put it that way. Because I wouldn't put it that way. Let's say, when you're sculpting a sculpture, you look at it.

(23:27 - 23:36)

You see there are certain parts where much work is needed. So, that is the way I look at my body. I'm sculpting.

(23:37 - 23:44)

So, I look in the mirror. I see that there are extra sediments of fats here. So, probably, I should work more on that.

(23:45 - 24:01)

So, yes, that is. I never tended to compare it with out of consciousness, I guess. So, how do you think cultural norms in India impact the way men view their bodies compared to other cultures? So, we're talking about an Indian subcontext.

(24:02 - 24:04)

Okay. So, how people view. Okay.

(24:04 - 24:15)

So, he's like, okay, he's the perfect man because he possesses all this. I'm not only talking about body. We can also talk about mustache, beard, height, everything.

(24:15 - 24:20)

Even my mom tells me, you're a man. You have to look like a lion. Be a lion.

(24:20 - 24:23)

Because you're a man. You're a born man. So, you should be.

(24:23 - 24:37)

You ought to be a lion. So, that's what their expectations. The Indian parents, Indian moms or anyone's of their expectations of this ideal masculinity or the ideal male body image.

(24:37 - 24:45)

So, what do you think of it? Okay. The ideal male body image. Yeah.

(24:45 - 24:50)

The cultural expectations. We are talking about in Indian subcontext. Okay.

(24:50 - 25:02)

Where, you know, like how people, our cultural norms, we can say cultural norms. Okay. Let's say we read something or this is how we have been brought up.

(25:02 - 25:08)

Okay. This is the cultural expectations norms of how men should be. Okay.

(25:08 - 25:32)

So, how does it impact you? I'm asking how these norms, these cultural norms impact you or your perspective to view your body or when compared to other cultures. We can compare our culture with the Western culture. How it is different? How the perspective of male body image differs? Okay.

(25:33 - 25:39)

Where do I begin? It starts from the hair. Right. Once a man goes bald, that's it.

(25:40 - 25:46)

They may say, what is it in your hair? Nobody cares. No, that is not true. We all know that.

(25:46 - 25:54)

That is not true. Not at all true. So, from the hair, especially to men, from the hair it begins.

(25:55 - 26:10)

Hair, the way you, even your voice. If you have a, they even call it, if a man possesses a very soft and sweet, I don't know how to describe it. Delicate feces.

(26:11 - 26:16)

Yeah. Yeah. A kind of particular voice, they will be discriminated.

(26:16 - 26:28)

Even though he can be, you can see the comedies in Tamil movies as well. The man will be, you know, huge and his voice will be very spewing. It's like spilling.

(26:28 - 26:41)

And he will be discriminated. So, what is the point of having all those huge arms and, you know, looking muscular? With the fraction of voice, you know, you lost it. You lost all the reputation.

(26:42 - 26:58)

And all the same with the hair. Also the beard. Once you, as brought up from a typical Indian family, I am expected to keep my mustache always.

(26:59 - 27:06)

If I remove that, there will be consequences. So, I cannot make them to understand. At least I can convince my parents.

(27:07 - 27:19)

But I cannot go on convincing, you know, I cannot go on explaining to the others. Even the people from the neighbors, you know. What happened? It is easily noticeable, man.

(27:19 - 27:34)

It is easily noticeable. If you take off your mustache and if you walk around the street, what happened to him? Why, why, why, why, why he is like this? Why he is so different? Why different? I just took my mustache off. So, yes, everything, everything has an impact.

(27:34 - 27:49)

And neck, there are people in the fitness world who expect you to have a broader neck. So, they keep some pressure on you. Everything is good.

(27:49 - 27:55)

Your shoulder is good. Why, why, why, why your neck is not? I do not care. That is it.

(27:55 - 28:03)

But the expectation still goes on. Okay. Speaking in a perspective of cultural representations.

(28:05 - 28:31)

One is the diet we follow. The diet, what you can never explain to the typical Indian parents about your nutrition diet. Because we as from their childhood, they had been following certain type of foods which are rich in carbs.

(28:31 - 28:45)

For example, rice. Rice is rich in carbs. So, what I cannot go and tell them that you have to reduce the rice and increase the vegetables, increase the veggies, increase the meat, meat intake.

(28:46 - 29:06)

You cannot do that. The immediate answer will be, why do you behave like a white man? Why do you behave like a Westerner? Be, be, stick to the culture, stick to our Indian

culture. But what really happened is that this is not the food we follow in the, in the actual ancient period.

(29:07 - 29:15)

So, I cannot, those things you cannot explain to them. If they say you have to abide or else no food for you at home. So, that is it.

(29:15 - 29:32)

And diet is one thing. And the main body, yes, hairs, hairs on your body. So, you are not, we usually, we boys or men are not allowed to remove our body hair.

(29:33 - 29:55)

If you shave off your body hair, I wanted to shave off my body hair to pose in the mirror to check my fat sediments and where I need more work. So, with hair, it is, it is not clearly, the definitions are not clearly visible. So, I would like to, but I am not allowed to.

(29:56 - 29:59)

If I do that, I cannot roam around the house.

Okay, the next question. Do you think that there is enough awareness and support available for men who struggle with body image concerns? No, I guess no, because this is the point, this particular point, even I am confused what is true, what I have to follow. Yes, even though I have a very, I am very interested in this, keeping my body fit and healthy.

I do a lot of research, but not everybody is up for the research. They just believe whatever they are being heard. There are people, because they don't want to spend time doing all the research and find out anything.

(0:55 - 1:25)

They believe that this particular brand is good and just jump into it and take it and start working on it. So, a proper guidance, no. A proper awareness, definitely not.

But there are awareness, I don't know whether to believe it or not. So, yes. Because, you know, let's say you go and switch on the TV, you find some cancer awareness, some disease awareness.

(1:25 - 1:58)

Even governments, if you travel on the road, you see these big banners, cutouts, that is, you know, like endorsed by the governments. Awareness. Okay.

Even for pregnancy. Okay. Mothers, women.

Okay. It's about, it could be about periods and everything. It's always about women and their health.

If you switch on the TV, you see the mother's care, the health care, the baby's care. Okay. And even issues with their body concerns, you know, after pregnancy, they put on a lot of weight.

(1:58 - 2:05)

So, it's always about, you know, counseling or awareness or support. It is dedicated. They are solely dedicated to women.

(2:05 - 2:39)

Women, yes. Yes. So, you don't think there are enough supports available, right? No, definitely not.

And even you can take, just go and watch the TV and how many ads you see, you know, related to men and their health, their fitness. Nothing. Even, for example, there are health drinks, which again, as I said, which again, which again represents the health condition of the body of women.

(2:40 - 3:26)

After pregnancy, they might have a weak bones and everything. So, that taking this particular type of health drink, you know, strengthens them. Even men do face those sort of things.

After the age of 40, everybody needs to do a body check. It is very basic. But how many of the advertisements, you know, encourage men to go for a body checkup? Because men are expected to be healthy.

That's it. You are a man, you have to be strong. So, yes, that is also, this is one way of seeing, you know, this is my way of seeing the lack of these advertisements and awareness is because the society indirectly expects or indirectly mentions that men have to be strong physically.

(3:27 - 3:41)

You are expected to be strong. Let's say if you carry a bag and you say that your hand is aching, you are a man, right? How can you say that your hand is aching? You have to carry it. That's it.

(3:41 - 4:25)

Yeah, so the reason and yeah, I don't find it. So, what do you believe are the most effective strategies for promoting body positivity and challenging harmful stereotypes about male body? Like you have said just now, you know, the stereotype about men feeling the pain, but they are denied the basic, you know, the support. So, the basic consideration, if you feel painful just because carrying this heavy weight, you don't need to.

So, there is not even those words uttered from the other people. So, these are stereotypes because just because you are a man, you have to tolerate everything. You have to carry the weight because you are a man.

(4:26 - 5:52)

So, what are the strategies that do you believe that could promote body positivity? First, what is body positivity? We have to have a, even I am confused because being fat is not body positivity. You know, you have to hurt yourself so that you can be free from diseases. Nothing is gained out of pleasure.

There is a pain in everything. So, as you said, certain words are not uttered to men. No, men are trained not to expect those words.

That is the issue here. The problem is not uttering those words. No, men are expected not to expect those words from the others.

Do you get it? Yes, yes, yes. You cannot expect someone, as a man, you cannot expect someone to utter you or fat you that, okay, it's okay, your arm is hurting. So, you can just leave it.

No, you cannot expect that. You are trained that way. And also, even I believe that in order to make yourself stronger, you have to go through a certain thing.

(5:53 - 6:47)

So, we men believe that, I mean, men like me, in the sense, men who grew up in an environment like me, we believe that if I carry this weight and if it is hurting me, if the groceries are hurting me now, it will not hurt me if I continue to do this for the next 10 days. By the 11th day, I can carry more. So, this pain is temporary.

So, you can see the confusion in me, right? I'm confused in a state whether it is okay to expect certain things, certain good words from the other or is it a path for me to be better? I'm confused in a sense. And this confusion is being manipulated by the other groups, other corporates and everything. So, we are very much confused, I would say.

(6:48 - 7:24)

So, what was the question again? Yeah, what are the strategies you can employ to promote body positivity? So, initially, you were saying, when you're fighting, how to hurt yourself to be healthy. I mean, you're talking about the process of going to the gym and reducing all the unwanted fat. And maybe it's not about building your body.

It's about becoming healthy. That's what you were saying. But to attain the goal, you have to hurt yourself in the process.

(7:25 - 7:38)

But yeah, but my question is about body positivity. Yes, fine. But it has a long way to go, right? Even if it is for two months, three months or one year, two years, they're still fat.

(7:38 - 7:51)

They're working out. I just know, not just within 24 hours, overweight, oversized person will become a muscular person. It takes its course.

(7:51 - 8:11)

Okay, what I'm saying is, okay, so to promote body positivity, yes, but that person is still oversized. Yeah, you have to come through all this. But yeah, first he has to accept himself, right? Okay, I'm oversized.

(8:12 - 8:29)

Okay, that is also a positivity. Okay. Don't you agree? Yeah, that's the problem.

That is why. This is my body. I accept it.

Okay, maybe I'm unhealthy. I need to work on it. Okay, again, so instead of that, okay,

now I'm so fat, nobody likes me.

(8:30 - 8:39)

Okay, I have to impress others. I want to impress others. Because I don't live for myself. I want to live for the society. I want to live for others. Okay, so I really need to go to the gym.

(8:39 - 8:51)

And you know, it's not about being healthy anymore. It's about being attractive and attracting others. Okay, so that is not body positivity, right? So you don't have to hurt yourself to be that person.

(8:52 - 9:31)

You can hurt yourself to be healthy, but before you become healthy, okay, so you are in your own body. Okay, but the feeling you should feel about yourself, I think it should be positive, right? Don't you think so? That was my intention. One thing, what I do, whenever people come to me and ask for, you know, whenever they went out on their body and their discretions, the one thing I would say, whenever people are in the process of progression, trust the process.

(9:32 - 9:40)

This is what was taught to me as well. Trust the process. Even a degree, even to complete a degree, there is a three year minimum requirement.

(9:41 - 9:57)

So nothing is going to be attained, you know, immediately. For the ultimate goal, you have to trust the process. You may not see the difference, you know, because since you, let's say you had been unhealthy for the past 10 years.

(9:58 - 10:06)

You ate all the junk food and you had been unhealthy for the past 10 years. You cannot expect a night change. We know that.

(10:07 - 10:50)

And in order to, this is the place where the media and the businessmen and everybody jumps in. They say that you use this, use this particular product, you will see the result

within 24 days. There is no such thing.
You have to trust your own process. You don't have to know the faith you are putting in certain chemicals and steroids. Please put the faith on me.
This is this is some kind of awareness which have to be made these days because it is OK. It is OK. It is OK that you, you, you, you, you, you had been unhealthy all these days.
(10:50 - 11:21)
But the thing we have to note is that you decided to change yourself by this day. So it is definitely going to take six to eight months to see some, see some natural transformation. If you're going by the abide by the natural way.
So you have to, we have to make it. No, we have to normalize this transition period. So so that so that this is the time where the people are being manipulated.
(11:21 - 11:33)
So why do you have to. It is very much similar to this Siddha medicine and Western medicine. If you take Siddha medicine, the curation, no curation, not the curation period.
(11:34 - 11:45)
The cure, the cure will be a bit delayed, but with no side effects. It is what it is, what we believe. But if you take Western medicine, the cure will be happen within two days.
(11:45 - 12:30)
So that is why. But we all know that Siddha in certain ways, it is always much better than the Western medicine. We people believe that.
And also there are certain proven scientific facts as well. Why do you waste your money on medicine when you have everything in your food? So that is why. Same thing. Everything takes time. And don't don't let people to manipulate you. So I built faith. What I would do is that for body positivity, I built faith in people. I built faith to trust their progress. So if they have enough faith, they will have enough courage to stand up for them.
(12:30 - 12:43)
Whenever people say that that you're fat, you're still going to the gym. Why are you still fat? I'm in the progress. You don't have to care about their perspectives when you're

concerned about your own health.

(12:43 - 13:56)

Because you are going to the gym not to impress a particular person, but to impress yourself. So, yes, that is one way of putting it. One last question.

From your perspective, what policy changes or societal shifts could help address the gaps in support for men dealing with body weight issues? So what are the policies that you can suggest or societal shifts that could help address the men who are dealing with body weight issues? It could be educational policy or any other government policy. I would say that since the government is very much on, we know that the government has very good advertisements on these cancers and pregnancies, regular checkups on pregnancies and regular checkup for breast cancers. But I would also request the government to do the same for men as well.

(13:57 - 14:26)

For policies, a government cannot compensate on the education, food and health of a community. So only when it comes to health, I believe it is mostly on women's health. So the government has to initiate some policies which should be focused on men's health as well.

(14:27 - 14:56)

Also, the awareness through the influencers, it is not always about the money. Because there are men who are ready to give up their increasing level of testosterone for a bit of hair on their scalp. At what cost? That is the question that is being asked.

(14:57 - 15:26)

Why should you give up certain things to get certain things? The whole thing is completely collapsed and confused in a certain way which even people like me are struggling to find the right path to get out. So we believe that this is the right path and we stick to it and we move on it. And definitely someday there will be a research paper and they say no, this is not, that is the path.

(15:27 - 15:53)

So this confusion, to avoid these confusions, to clarify these confusions, doctors have to

come forward and give us certain guidance. Not at the cost of our own health or money, just genuine guidance. And it is being in progress these days.

(15:53 - 16:07)

You can see there are a lot of doctors who are concerned about men who go to the gym. And also there are fitness trainers who correct the forms. You have to stick on to a certain form to avoid injury.

(16:08 - 16:19)

So even those are being corrected these days, but it is always not enough. So much more have to be done. So those things, it is a good initiative.

(16:19 - 16:35)

I want that to be continued in a bigger spectrum. What about educational institutions? For example, even for people who are addicted to alcohol, they have these rehabilitation centers. They have these rehab centers.

(16:35 - 16:50)

And also they have these support groups. You would have watched it in western movies where they have these form circles and talk about it. Why they are addicted, why they got addicted to drugs or alcohol.

(16:51 - 17:11)

Even for cancer patients, they have these support groups. Do you think that could be implemented in schools or colleges? Because we have all these drama clubs, arts clubs, music clubs, dance clubs and everything. We have clubs for almost everything nowadays.

(17:14 - 17:38)

Since this is a very important thing for any human being, do you think that it could be implemented in educational institutions? Yes, I believe there are already reforms on that. We have PT classes, but PT classes are vanishing these days. You can see that in school education, there are no more PT classes.

(17:39 - 17:54)

For the namesake, they have PT classes. And those classes should be taken into serious

consideration. Because you never know that a particular student might need those relaxations.

(17:55 - 18:01)

Exercise is another form of relaxation. Sports is another form of relaxation. It varies from person to person.

(18:02 - 18:13)

So those classes have to be taken into serious consideration. And it cannot be dodged. It should not be dodged.

(18:13 - 18:28)

In education, yes, they have very good reforms on fitness. But exclusively for men, that is a question. Maybe that should be included in the topic while doing PT exercises.

(18:30 - 18:46)

Since they have separate classes for women, even during my college days, they used to take women out separately. They get instructions on their body and everything. And women never had it.

(18:46 - 19:00)

Because there is an assumption that women get to know things by nature. I don't know what's going on there. Nobody is going to talk about it.

(19:01 - 19:10)

Nobody is willing to talk about it. That is one thing. It is through friends or it is through our seniors.

(19:10 - 19:31)

That is how we get the knowledge. And you can see, if the knowledge is attained through friends or by seniors, there might not be any proper guidance. So yes, a proper guidance, including a proper guidance in the academy, is much appreciated.

(19:33 - 19:36)

Thank you. You're welcome.
