# Supplementary material for: “An interpretative phenomenological analysis of male body image through the lived experiences of men in India”
Source: BMC Psychol. 2025 Jul 1;13:714. doi: 10.1186/s40359-025-02963-y (PMC12219639; doi:10.1186/s40359-025-02963-y)
Supplement: Supplementary file 5 — Supplementary Material 5. [file 40359_2025_2963_MOESM5_ESM.pdf]

(0:03 - 0:21)

Okay, so I would like to tell you the idea of this paper, which I'm writing. Do you know the concept of body image? You can share, so please be free. Though this is a formal recording, you can be free.

(0:23 - 0:50)

Can you just tell me about body image? Body image is how we perceive someone's or our own body. So I'll just tell you about the name of my study. So this paper is called an interpretative phenomenological analysis of male body image through the lived experiences of men in India.

(0:51 - 1:22)

Bridging policy and practice for gender equality and sexual diversity. So in simpler terms, so we are just going to, you know, so you know how to do research, right? All the structures and procedures in research. Yes, yes.

Okay. So you know this methods and methodology in research part? Methods, methodology. Okay, if you're going to conduct research, we need to have a method.

(1:22 - 1:30)

Okay, it could be qualitative or quantitative. You might have heard the survey methods and all in quantitative. Okay, so this is an analysis method.

(1:30 - 1:37)

So you might have known this thematic analysis. If you're going to understand something, we can analyze through the themes. Okay, if you're watching a movie.

(1:38 - 1:49)

Okay, so you might, you know, come across these concepts of love, friendship, betrayal. These are themes. So we are going to analyze the movie or a novel through the themes.

(1:50 - 1:59)

It is the same. Okay, it is as same as the, you know, thematic analysis in a research paper. Okay, so this is called interpretative phenomenological analysis.

(2:00 - 2:11)

Meaning, so when you, so you would have had some experience in your life. Okay, so

that is your first interpretation of that phenomenon. So something happens, you see it, you experience it.

(2:12 - 2:24)

That is your first interpretation, right? So when you narrate that story to me, that is the second interpretation. Okay, then I'm going to write your experience. I'm going to interpret your experience.

(2:24 - 2:35)

So, first, second, third interpretation. So this is how, you know, the study is going to analyze the experiences of the participant. Okay, because, you know, it's not the firsthand interpretation.

(2:36 - 2:44)

You already interpreted in the first, I mean, in the first place. Then when you are narrating, that is second interpretation. When I'm narrating, that is third interpretation.

(2:44 - 2:55)

So this is just, you know, one of the methods to analyze. Okay, fine. So I have a few questions regarding, related to body image, male body image.

(2:56 - 3:14)

Okay, when you say body image, it's usually, you know, concerned about women, right? It's always, you know, women's body. Okay, so what problems they face in the society, it's always women. So even when you look at the research part, it's usually, the research is on women.

(3:15 - 3:25)

So there is no much existing literature about men. So that's what this study is going to all about. Okay, so this is the basic understanding I want you to know about the paper.

(3:25 - 3:32)

Okay, fine. I just want to get you to the questions, a few questions. Okay, simpler questions.

(3:35 - 3:39)

If you don't understand, you can ask me again. I'll just put it in simpler terms. Okay.

(3:41 - 4:03)

How do societal expectations of masculinity influence your perception of your body? Okay, so there are certain society's expectations, right? How a man should be, that is called masculinity. How a man should be. Okay, so how these expectations influence your idea of, you know, your perception of your body? What do you think? My body.

(4:03 - 4:15)

Okay. Society wants us to be not that fat. Like 25% body fat is a bit too much.

(4:17 - 4:30)

Around 22, 21, it's like fine. Then as we go lower, 15 is like bodybuilder type. And below 15, I would say it is like unhealthy.

(4:31 - 5:04)

For me, I would say, since my body fat is a bit more, I'm kind of unsatisfied with my body. So how do you think that, so every time you experience this, or from the external force, or it is just, you know, whatever you're having, you're having internal conflict or so others are affecting you. I'll say, normal regular people that I meet won't tell me that I'm fat.

(5:04 - 5:21)

The people who tell me that I'm fat are the people who are close to me, people who I go to the gym with. As we want progress, they'll criticize my physique. Okay, so you think that they're criticizing you because they want you to get into the progress.

(5:22 - 5:36)

Yeah. But do you think that's a healthy criticism? Yeah, I mean, it's healthy. If it gets me to work more, then it's healthy.

(5:37 - 5:55)

What if there is no one to criticize you? What happens then? Then I don't think I will actually be improved. Okay, you can put it in other words. You don't want to be improved or you don't want to be healthy, body conscious.

(5:56 - 6:13)

Let the healthy conscious be aside. So if no one is there to criticize you, you won't focus on your body, right? That's what you want to say. It's just because of the society or the

external force, you want to improve your body.

(6:15 - 6:26)

I'll have to think. I'm just confusing you. See, you gave me an answer.

(6:27 - 6:51)

Okay, if my body fat is causing hindrance to my daily life, then I would reduce it without any external motivation. But if it's not causing any difficulties to me, then I would stay comfortable in that level itself. Next question.

(6:52 - 7:15)

Can you describe any subjective experiences where you felt pressure to conform to certain physical ideals associated with being a man? I'll just put it in simpler words. Do you know what physical ideals are? Let's say we have bodybuilders. When you go to Instagram, Facebook, or any other social media, you see someone who has a very built body.

(7:15 - 7:43)

Or any other actors, mainstream actors, who have six packs and very good build. So when you look at them, do you feel any pressure? Any subjective experience, personal experience, where you want to be like them? So that you can be considered a man. Do you understand? Not to be considered a man, but to be considered attractive.

(7:44 - 8:01)

For that, most of the people I know will be chasing at least one. They'll consider one person as their goal physique and try to work towards it. Even if they are steroids and unhealthy level muscular, they'll keep them as their ideal.

(8:03 - 8:15)

Do you have any personal experience of having this physical ideal? It could be anyone. You just want to be like that person. You want to build your body like that person.

(8:17 - 8:32)

I do actually have an ideal. When we are kids, we'll think we'll become like Arnold, Big Jackman. Then when we start lifting, we'll realize it's hard.

(8:32 - 8:43)

And our expectations will reduce. And we'll eventually find someone who is naturally, natural level fit. And we'll set them as an ideal.

(8:44 - 8:59)

I have an ideal personality. Nice. In what ways do you think media representations contribute to shaping male body perceptions in our society? In the context of India, in our society.

(8:59 - 9:19)

Let's now talk about the world. Let's talk about Indian setting. What do you think the role of media plays in shaping your perspective of male body? Actually, Indian bodybuilders are not open about steroid abuse.

(9:20 - 9:33)

And when they post their pictures, most of them are misguided. They'll think this is naturally achievable and they'll keep on working out. Then they'll end up having body dysmorphia.

(9:36 - 10:07)

Perfect. So when you come across any social media or reels, images, videos, how do you think that affects you? What are the influences that these media play in your life? It actually motivates me. But if the person I see has too much neck, too much vascular, too much veins and disproportionate muscles, then I can understand this steroid abuse.

(10:08 - 10:27)

So I'll just ignore it. Don't you think that's a preconception? What if we just naturally built it? No, no way. Either he is genetically gifted or he is a steroid abuser.

(10:29 - 10:48)

So when you said it has a good influence on you, when you said social media had a good influence on you when you came across all these videos, reels. But not in some way that it affects you. It just forces you to be like that person, like that body.

(10:49 - 11:05)

Don't you think that it's a good influence? Or do you think it also has a negative influence on you? Yes. Sometimes I feel like body dysmorphic. But then I'll be like, in two

years, I'll be closer.

(11:05 - 11:18)

In one year I'll be closer to achieving that physique. Long term goals. So there is this constant heaviness on your shoulder from the media.

(11:22 - 11:37)

I was having this conversation with one of the other participants. So that person was saying, even if I open my social media, it's always the bodybuilding. Always the gym people.

(11:37 - 11:55)

There isn't one time if I'm not opening Instagram or anything, there won't be any bodybuilders. There will be definitely at least one. Someone who posts a picture or status from the gym.

(11:55 - 12:10)

There should be at least one every time I open the social media. So this person was saying, this participant was saying, since this gym trend is going on now, today it's the trend. Tomorrow it could be different.

(12:11 - 12:21)

If tomorrow is all about having an oversized fat body, I will go with it. Today it's a gym trend. So I want to go to the gym and build my muscles.

(12:22 - 12:29)

Because it's a trend now. Today it's a trend. If tomorrow it's all about having this fat body, I will become fat.

(12:29 - 12:36)

That's what he said. He said that. That's not a healthy way of thinking.

(12:37 - 12:44)

Okay. Okay, I agree. At least you didn't say that's not a healthy way of thinking.

(12:45 - 12:49)

I mean, yes, I understood. Fine, fine. So you are completely against it.

(12:51 - 13:05)

I will never stop going to the gym. If the abs physique goes out of trend, then it will be the bulky physique. I will be fat with muscles.

(13:07 - 13:27)

So you are hitting the gym now in college? Going to the gym now? How many people are going? Who will I meet? Everybody is going to the gym. How much people they could afford? I don't know. Okay, next question.

(13:27 - 13:50)

Have you personally experienced Have you personally experienced body dissatisfaction or concerns related to muscle dysmorphia? Yes. Can you just tell us? You can talk about whatever that comes to your mind. Incidence of muscle dysmorphia.

(13:54 - 14:08)

Before I started going to the gym, I was skinny fat. I mean, I had fat and I didn't have much muscle mass. Then when I started, I actually started going to the gym to reduce my weight.

(14:08 - 14:16)

I was 75 kilograms then. Then within 6-7 months, I gained 20 kgs. I gained muscle, but I didn't lose fat.

(14:18 - 14:29)

Okay. I have never cut down below 20% body fat. I haven't seen myself in that state physically ever.

(14:30 - 14:44)

So that is something dissatisfying. Because of academics and exams and all. When I say muscle dysmorphia, it could be a disorder.

(14:44 - 14:51)

It's a disorder. There is this constant comparison. We always feel short of muscles.

(14:52 - 14:56)

We always go to the mirror and check. Always feel that we are small. We are built.

(14:57 - 15:03)

We are somewhat built. Somewhat defined. We have a defined muscle.

(15:03 - 15:10)

We always feel we are inferior than others. So I don't have enough muscles. There is this constant comparison.

(15:11 - 15:37)

Whenever you cross or whenever you look at the mirror or other people. It could be a mental condition. Do you think you have ever had these thoughts of feeling inferior? Or constantly comparing to yourself or to others? Not only about muscles and appearance, but in gym culture.

(15:38 - 15:46)

Inside the gym, people usually compare their lifts. 80kg bench press, 200kg deadlift. They will compare with each other.

(15:47 - 15:56)

People with less numbers will feel dissatisfied. I used to feel that. But then after some time, after watching some motivation.

(16:05 - 16:10)

Okay. I have heard this also. I have personally experienced this.

(16:10 - 16:24)

I started with 2.5kg dumbbells. Then it got to 5kg. Then 7.5kg. But I couldn't lift more than 7.5kg at some point.

(16:26 - 16:31)

I was 82. From 62 to 82. I put on weight during the Covid.

(16:31 - 16:43)

But I couldn't still lift more than 7.5kg. But people who were less than my weight. They could lift 15-12kg. That made me constantly worry about myself.

(16:43 - 16:50)

I am not fit even if I go to the gym. And also one more thing. When I see people younger than me.

(16:51 - 16:59)

15 year old boys on Instagram who will be lifting more than me. I should have started

earlier. That's true.

(16:59 - 17:08)

Even I have seen 10 standard boys. I have met 12-11 standard boys who have abs. Who have packs.

(17:09 - 17:17)

I can't believe it. I studied in a hostel. I stayed in a hostel in my 12th grade.

(17:18 - 17:23)

Not 11th. Just 12th. But I remember people having these dumbbells.

(17:23 - 17:26)

Constantly working. But they didn't have these packs. But they were fit.

(17:27 - 17:32)

Whatever we consider fit, they were fit. They had this fit body. But nowadays it's more than fit.

(17:32 - 17:38)

It's moving beyond the fitness. It's having the build. Muscular, beef.

(17:38 - 17:49)

So that looks all matters. I also noticed one thing. Have you watched the movie I, Tamil? Yes, of course.

(17:50 - 18:06)

Yes, I watched it. In that movie, Vikram's physique, if we look at it now, we will be thinking how did he even enter the competition with that physique. You are asking me something.

(18:07 - 18:17)

How did he enter the competition with that physique? No, I am telling. We will be wondering how did he enter the competition with that physique. Now that we are looking at it.

(18:18 - 18:26)

Yes. That's true. Because I think most of them had more muscular physique than him.

(18:27 - 18:49)

They had muscle but they were not defined. Now if we go to any random competition, any district level competition, the people who are competing will be steroid abusers who have big muscles and much defined abs. So do you think it's more of steroids now? Not natural muscle building? So people are consuming steroids.

(18:50 - 19:04)

Most of the competitions, whenever I talk to my trainers about this competition, they will say all of them are steroids. Even if they take steroids, they are open about it. My trainers here in my land, Kerala.

(19:05 - 19:14)

Okay. That's a shocking statement. I was going to ask you if you had any personal experience with that.

(19:14 - 19:19)

I mean, with your friends or someone. But yes, I understand. I believe you know.

(19:20 - 19:31)

Okay. Next question. So how do you think cultural norms in India impact the way men view their bodies compared to other cultures? So we are talking about cultures in Indian context.

(19:32 - 19:46)

So when I say body image, it's not just in physics. We can even talk about moustache, beard. So in certain cultures, even in Tamil Nadu, my mom used to tell me, you are a lion.

(19:46 - 19:57)

You are a man. So when she says that, she emphasizes this sign. You are a man.

(19:58 - 20:09)

You are masculine. Power. So we have this culture, right? So when you compare this culture to the Western culture, they don't usually have this beard, moustache.

(20:10 - 20:24)

Okay. But now, in Tamil Nadu, if you don't have this moustache, I'm not sure about other states, but if you don't have moustache, beard, they constantly criticize you. You don't

have this.

(20:24 - 20:30)

So only men have. If you don't have, you are a woman. So that's what people will tease you.

(20:31 - 20:37)

It happens. I've seen it. So you don't have to only talk about moustache and beard.

(20:37 - 20:49)

Body image in particular. So what do you think? How it is different from our culture to the Western culture? The body image. You have to be like this.

(20:49 - 21:00)

Normativity. Norms. Do you understand? About moustache.

(21:07 - 21:23)

Whenever someone without moustache comes to argue, the people here will say, I don't want to argue with someone without hair under their nose. Okay. Fine.

(21:23 - 21:37)

So they just use that as a shield. Most of the people, like young teenagers, are struggling to grow beard. Now beard oil, such things are gaining popularity.

(21:38 - 21:53)

Just to, just so they can pretend to be more masculine by growing facial hair. Okay. Why do you think pretending to be more masculine? Actually, that doesn't make you more masculine.

(21:54 - 22:04)

Masculinity comes from our actions, actually. Okay. Can you define actions? If you want me to stop asking these questions, I'll stop.

(22:04 - 22:16)

Don't worry. I just want to know because you're giving more answers. Actions as in taking care of the people we love.

(22:17 - 22:24)

That is one of the masculine actions. Providing for the people we love. Okay.

(22:25 - 22:45)

Understood. So have you ever felt judged or stigmatized based on your physical appearance? Judged? No, I haven't been judged. Yeah, I haven't been judged.

Not now. Not now. I used to be judged earlier.

(22:47 - 23:15)

Because now you're in college. So people who are very sensible, they won't do all these comments, I guess. I hope.

Okay. I think we have evolved. Okay.

So yeah, can you just give me some, you know, first-hand experience of how you felt when you were judged or stigmatized based on your physical appearance? Regarding your body image. I have to think. Sure.

(23:16 - 23:46)

Take your time. Yeah, when playing sports in school, this is actually a justified judgment because whenever someone is coming to play, asking in our football team, they will see their appearance. If they're fat, they'll just assume they are bad at sports.

(23:50 - 24:03)

So you've personally felt that? Yeah, I have. Oh, you were good at football? When I was 10 years old. When you were 10 years old? Yeah.

(24:05 - 24:25)

Do you play football now? No, I stopped playing after I got into an injury three years ago. Because I know this, you know, Maldo is having weird obsessions with the footballers. I know.

(24:25 - 24:31)

I was actually not that good at football. It's fine. You can try.

(24:31 - 24:44)

You don't have to be good at something to play. Okay, good. Do you think there is enough awareness and support available for men? If you look at women's support, there are many.

(24:44 - 25:04)

Regarding health issues, pregnancy, motherhood, even body image issues, women have support. Do you think that men have enough support about their body image issues or any other disorders? Any support available? Because they are struggling, right? No. No? Okay.

(25:05 - 25:08)

Fine. No. Okay, fine.

(25:08 - 25:28)

If they feel disappointed in their physique, they'll go to the gym. When they go to the gym, they'll keep comparing with others and the problem will increase. So do you think that is a form of support available? Okay, negative support.

(25:28 - 25:44)

If I feel body dysmorphic or muscle dysmorphic, instead of getting the support, I'll get the gym support and I make the problem a little bit severe. That's what's happening. Okay.

(25:45 - 25:48)

Fine. That's a good answer. Okay.

(25:49 - 26:01)

Fine. So how do you think traditional notions of masculinity affects men's mental health, particularly body image issues? So yeah, notions, traditional notions. So we have this traditional notion set.

(26:01 - 26:10)

So I think I have already told you about how a man should look like, which is conceived by people here. Okay. So this is how a man should look like.

(26:10 - 26:24)

This is the quality of masculinity. How do you think it affects the mental health of men, especially regarding their body image? It affects men's mental health. Okay.

(26:24 - 26:54)

These traditional points, traditional perspectives in India. When I say traditional perspectives, in Indian perspective, how do you think all these ideas of masculinity, how

a man should be, affects the mental health of men regarding their body image? I hope you understood the question. It affects the mental health negatively, but I don't know how to elaborate.

(26:56 - 27:14)

Okay. It's okay. So what do you believe are the most effective strategies for promoting body positivity and challenging harmful stereotypes about male bodies? Just effective strategies to promote body positivity.

(27:15 - 27:24)

What do you think as a person? Bodybuilders should be open. Bodybuilders should be open about steroid use. Okay.

(27:26 - 27:42)

How do you think that promotes body positivity? So that they can feel positive. People will set realistic standards for themselves. Okay.

(27:42 - 27:56)

I understood now. Okay. So people who misconceive, you know, who take steroid abusers as their ideals, they will get to know.

(27:56 - 28:05)

Okay. So these are not our real ideals. That's what you're meaning to say, right? So if they're open about their steroid conception, people won't go after them.

(28:05 - 28:09)

Okay, fine. Anything, any other strategies? Yes. I am approved of it.

(28:09 - 28:13)

Okay. I understand it. Any other strategies? Promoting.

(28:13 - 28:17)

When I say promoting. Comparison with self. Okay.

(28:18 - 28:21)

Okay. When I say promoting. Yeah, it could be.

(28:21 - 28:26)

Yeah, very good. Okay, it could be like it could happen within ourselves. Body positivity.

(28:26 - 28:33)

I don't have to like spread the positivity. Okay, we can spread. Okay, I can, you know, spread this body positivity for myself.

(28:33 - 28:42)

Yeah. Any other strategies? What can we do to promote the body positivity? Comparison with past self. Past self rather than others.

(28:43 - 28:46)

Comparison. Past self. Comparison with past self.

(28:48 - 28:59)

Can you elaborate? I don't understand. Like yesterday. Yesterday, if I could lift 10 kgs and today if I could lift 12 kgs then that's progress.

(28:59 - 29:07)

Just seeing progress and not comparing with others. Not comparing with others progress but ourselves. Our own progress.

(29:08 - 29:11)

Okay. Okay, I understand. Okay.

(29:11 - 29:29)

Good. So what do you want to say about people who do not who do not hit the gym? How can they you know, promote body positivity? If they are happy with their body and it's fine with them then it's fine. Okay.

(29:30 - 29:41)

Good. If they are okay being judged by others if they are okay being judged by others then it's fine. Okay.

(29:42 - 29:45)

That's a good response. Okay. Last question.

(29:46 - 30:14)

From your perspective which what policy changes or societal shifts would address the gaps in support for men dealing with body image issues? Again, when I say policy it could be any policy government policy or educational policy. Okay. How do you think

they can help us? Because you know when someone so you have this cancer campaigning right? So you would see all these you know banners from the or advertisements from government or not only or this even alcohol consumption.

(30:15 - 30:25)

Okay. People who got this liver failure liver cancer for consuming alcohol. You see all this from government ads.

(30:26 - 30:30)

Okay. Even if you go to theater you will see all this. Okay.

(30:30 - 30:34)

Advertisement. Okay. So they have these policies also.

(30:34 - 30:58)

Okay. So what do you think a government policy or any educational policy is needed? What kind of policy can we you know support this to support mental health health people who deal with body image especially men because it's always you know the support is available for women. Okay.

(30:59 - 31:14)

What do you suggest? What policy? It could be educational policy. It can be implemented in school or college also. What could be done? I'll have to think.

(31:14 - 31:30)

Okay. So because I was talking to one of the participants he was saying you know we could have the support group. You know rehabs right rehab people who who are addicted to alcohol they have this rehab centers.

(31:31 - 31:36)

Okay. Even in schools and colleges we have drama clubs and dance club art clubs. Okay.

(31:36 - 31:47)

So he was suggesting we could have a you know supporting groups. Okay. Which we can talk about anything something like that.

(31:48 - 32:14)

You can even talk about that same thing. If you're if you agree with it having a support

group in college school or you can say any societal shifts. Okay.

(32:14 - 32:21)

Okay. Or any societal shifts also societal shifts. That's also fine.

(32:22 - 32:26)

Yeah. No I can't think of it. Okay.

(32:26 - 32:29)

I'll give you a few examples. Okay. Even if you're not answering it's fine.

(32:30 - 32:38)

Okay. If you just turn on your TV you will see all these advertisements. It also has something to do with media representations.

(32:39 - 32:58)

If you open the TV you'll see all this beautiful okay fair-skinned muscled TV actors endorsing some product that we are not going to buy. Okay. Let's say it's just coffee powder or something two rupees coffee powder and they will just endorse it with the ads.

(32:58 - 33:04)

They will just show the ads and endorse it. Okay. Even for a shampoo they're just going to wash the hair.

(33:04 - 33:13)

But again they will show you the ads six packs. It's not promoting anything. I think it's promoting body dysmorphia.

(33:14 - 33:15)

Right. Okay. Look at this actor.

(33:15 - 33:22)

Look at this model. He's just washing his hair but look at his abs. That's what we always think.

(33:23 - 33:39)

In that way. So what should we expect even in advertisements something like that. Can you think of for hair they can they can use some models who have good hair instead of

good physique.

(33:41 - 33:45)

Just good physique. Yeah. So that is a societal shift.

(33:45 - 33:47)

Right. Okay. Yeah.

(33:47 - 34:00)

So instead of just you know even they have this fake hair or even women. Okay. They have this fake and photographic not photographic Photoshop photoshopped hair which looks so flauntless and flawless.

(34:01 - 34:08)

Okay. They just flaunt it but it's not their real hair. But still they're just beautiful.

(34:08 - 34:12)

They just show it. Okay. That doesn't promote body positivity.

(34:13 - 34:18)

So this is we can take it as a societal shift. Okay. Also policy implementation.

(34:19 - 34:26)

Fine. Anything you want to say? Do you have anything to say? That's it. All together.

(34:26 - 34:29)

It's not about just leave all the questions. No. Yeah.

(34:34 - 34:37)

That's it. Okay. That's fine.

(34:37 - 34:43)

I'll just you know stop the recording. Stop.
