# Supplementary material for: “An interpretative phenomenological analysis of male body image through the lived experiences of men in India”
Source: BMC Psychol. 2025 Jul 1;13:714. doi: 10.1186/s40359-025-02963-y (PMC12219639; doi:10.1186/s40359-025-02963-y)
Supplement: Supplementary file 6 — Supplementary Material 6. [file 40359_2025_2963_MOESM6_ESM.pdf]

(0:00 - 0:48)

Okay, so just a moment. You don't have to give your name. Okay, we are going to change your name, okay, to maintain the confidentiality, okay.

So this is a voice-recorded interview and you don't have to mind it. You can be informal. Just forget that it is being recorded, okay.

So do you understand the basic idea of this study? So the study, you know, the name of the study is an interpretative phenomenological analysis of male body image in India, okay, the lived experiences of men in India, okay. So it is about bridging policy and practice for equality and diversity. So basically, the study, you know, aims to grow awareness of body image concerns.

(0:49 - 4:06)

Okay, you understand, right? Okay, so basically, this research paper is going to address the gap because when you look at it, it's always about, you know, females, female body, women's bodies, okay. That is always, you know, the topic of concern, okay. You understand, even after birth, before birth, okay, so don't body-shame the women.

It's always the females. There is not enough topic about men. There is not research, okay, enough research, existing literature about men's perspectives, men's body image, okay.

So that is what this research is going to address by, you know, touching upon the social expectations, the masculinities and the perceptions, body dysmorphia, all these concepts, few concepts. This is the basic thing and the methodology we adopted is an interpretative phenomenological analysis. That is, you are going to give me your lived experiences.

We are going to, you are going to tell me that, right? So I'm just going to interpret it, not just merely describe it, interpret what you have, you know, gone through, your experiences. That is basically, this study is all about, okay, and you understand, okay. I have 10 questions, okay, but we can, you can talk about anything even if it is not about the question.

Okay, shall we start? I just wanted to tell you something. Sorry? I just realized that it's

not relevant to your... Which one? Something about this. Yeah, it's fine.

Do you want to start like that? No, you ask questions. No, what do you want to say?

Okay, sure? Yes. Okay, okay.

So, first question, just can you come closer? Just be a little loud, okay? Okay. Forget this.

So, how do societal expectations of masculinity influence your perceptions of your body?

Societal expectations.

First, I'll have this inferiority complex, you know, like, have to impress somebody, like, you know how I am. I feel I shouldn't be like this. Why? It's because the societal expectations on me, like, I guess, you know, masculinity, I need to be big, I need to be, you know, just to impress society, just to impress people, I guess, like, yeah.

How do you think these are expectations? Like, I hear from people, right, you are a boy, you're a man, you have to be big, you have to be, I mean, from my family, from my, you know, not my mother, I can't say my mother, but my grandparents, you know, that generation, they expect us to be big, they expect us to be. Even my mom says, you know, like, you're a man, be a lion. Yeah, exactly, exactly.

So, not only, you know, the way you talk and speak, no, it also has to do with your body. That's what I meant, that's what exactly I meant. So, how does it influence your perception of your body? I just told, I have that inferiority complex, so I need to.

(4:07 - 4:53)

Need, so there is a need. Yes, sir, I need to improve it, I mean, of course, I'm trying to, but still, you know, trying to, like, have food, do gyms, do stuff, do, you know. But you're just 18, right? I'm 18.

18 in your first year, but why do you think that this all matters? You're just, you know, like, entering your, maybe what we call it, manhood, okay, and you're a student. Yes, like, why do you think that this all matters, really matters? I mean, I don't want to be like that in the very near future, but I guess now I have to start to, you know, build that. How to, why is there a how to? You're talking like a journalist.

(4:54 - 6:40)

Okay, I'll just, sorry, I'll just be, that's what I'll do. Okay, why is it how to? Because, you

know, even in the previous interview, there's always this word how to, how to, how to. Okay, why how to? Like, I told, to impress people, I mean.

Okay, understood. To impress people, not in the sense, like, yeah, to impress people, to impress. Basically, okay.

So, can you describe any personal experiences where you felt pressure to conform to certain physical ideals associated with being a man? Do you have any subjective experiences? I don't think so. Like, like, somebody would have criticized your body. That is your experience, right? Yeah, body shaving, I mean, we go through that, right? We go through body shaving.

You said you have no experience. Yes. Can you recollect something that would have just hurt you, affected you? When we are hanging out with friends, they make fun of, you know, how you look.

Like, how, what do they say? It's okay, just know. I'll tell you. I mean, by sight.

So, I have a roommate, so we casually talk, we casually, you know, roommates, we fight for small stuff. And then he says, I mean, he says in a positive way that I'm not hurting you only because I know you can't hurt me back. That is also body shaving, right? I mean, he says in a positive way, but still, you know, you know how I feel after that.

Yes. I mean, I'm not, he is not that. Yeah, I understand.

I understand that. But still, it's passively, it could passively hurt. Yes.

(6:41 - 11:49)

That was an experience. Okay. Anything else that you could think? Because, you know, sometimes, you know, it is actually a form of pressure, right? To conform to that idea of masculinity.

Also, not just physically, but even if, you know, even if a man cries, people can't take it. People say, you're a man, men don't cry, do not cry. We are like, we have to, again, we have to hide our emotions.

If we be really, like emotionally, if we, you know, like I have gone through this, I was crying for something, and then they told you, you're not a gentleman, you're just a boy. You are really, you know, express. We, you know, we can't be more expressive, we can't

be more emotional.

I mean. Because it has something to do with your masculinity. Of course.

I cry all the time. The last time, the last time I cried was two days ago, I think, by watching some movie. That's what I'm saying.

We are not expected to do that, for some reason. I don't know. It has nothing, like, emotions has nothing to do with gender, right? I don't know why we are not expected to cry.

Even crying is not only a physical, I mean, mental thing, it's also a physical thing, because you're crying, it shows on your body, it is visible. So, nobody, you know, accepts when you are mad and cry, when you pour out your tears. Okay, understood.

So, do you have any physical ideas, like where you worship someone, like an actor or, you know, you understand? Yes, of course. Like, can you talk about it? Like, let's say, if I watch some hero, maybe Shah Rukh Khan movies or any South Indian actors, Ram Chandra or someone, okay. So, they have very beautiful physique, very defined, okay.

So, I mean, yeah, of course, we do admire, but I don't feel like I have to be like him, you know, I don't feel like I should be like him, but still, I admire. I wish I were like him. It's not that I have to be like him.

I mean, it's nice if I am like him. It's nice? It's nice if I am also, like, if I also have the, you know, body like him. So, they're promoting, right? So, you have this ideal, okay, if I ever develop a body, I should develop a body like him.

Yes. So, we always have these ideas. Where do you find all these ideas apart from TV? Maybe you have any real-life ideals? Most of the influence after movies and that stuff. Okay, because, you know, sometimes, if I see any professor who is very fit, I just, I mean, the idea of fit, I mean, I just, okay, I'll tell you a few more things about my body image, okay, fine. Just a minute. So, in what ways do you think media representations contribute to shaping male body image perception in our society? In our society, the contribution of media representations? I feel it doesn't care much about, you know, like you told, the, how do I say this? The importance they gave to female that we are not getting.

See, nobody, you know, nobody cares about, you know, also, just, I mean, also,

whenever there's a crime news or something, we see, we see the female faces are blurred, but in men's faces, they don't do. For some reason, I still don't understand the reason behind that. Okay.

I know that is not relevant to this, but still, you know, doesn't care more about men. Okay. This I've, like, seen, like, number of times, whenever I see news, a female face will be blurred and then the man's face wouldn't be blurred for some reason.

Even, doesn't matter who, like, made the crime. Even if the female is the victim or the male is the victim, his face will be clearly visible. His identity, they're showing his identity, but then her identity, they are not.

Okay. So, how do you connect that with a body image? Yeah, this context I understood. So, how do you connect that with, you know, basically, shaping male body image perceptions? Like, if you, if you use social media, you use social media, you have Instagram and stuff, okay.

So, when you watch reels, you know, of bodybuilders, okay, because, you know, when I open my Instagram or Facebook, you see all the fit people, okay, what we call fit, okay. So, like, when you go, come across all these reels and stuff, how do you feel about it? The admiration we have, like I told, we want to be like him. Yes, it has an influence on our, you know, mindset, on our thoughts.

(11:49 - 12:44)

Does have an influence. It does have an influence. Yes.

So, have you personally experienced body dissatisfaction, which you already partially answered, okay. Yes. So, our concerns related to muscle dysmorphia.

Can you explain what it is? Yeah, just, you know, the lack of muscles, okay, like, just, you know, just with skins, covering the bones, okay, when you feel a shot of muscles. So far, I didn't have the need to use my, you know, physical, okay, you know, power, like, I didn't have to use that anyway, like, I was, I wasn't in a trouble to, you know, have, use that. But actually, you know, hypothetical case, what if, what if someone attacks me and I can't, you know, what do I say? So, this is, that's what we call dissatisfaction here.

(12:44 - 13:54)

Yes. So, we are experiencing something dissatisfied with our body, okay. So, when someone, maybe if you feel threatened, you cannot act out because of your body, right. So, when I say muscle dysmorphia, it's actually a condition, like a constant, it's a disorder, actually. It's a disorder, it's a condition that, you know, you constantly worry about your body, okay, being too small, that is muscle dysmorphia. Because I don't have the, you know, like, the presumed or, you know, the very traditional form of masculinity, masculine body, where I have to be, like, rigid, like, stuffed, beefed, okay, like, not broad, not having broader shoulders, not having the proper biceps.

So, do you have that dissatisfaction? Yes, yes, yes. Can you just briefly explain? I guess I gave you the answer. It's okay, just, okay, fine.

Like, when you see others, when you see people who have... You know, some of our friends, like, you know, Plashun from RBRS, he has, like, a really good, you know, physique. I mean, I don't know if you have seen it or not. He posts on Instagram.

(13:55 - 14:16)

Every day, even today, I think that's how I woke up. We also kind of want to be like them, you know. How does it make you feel, like, someone has that, but you don't have? Oh, I don't... What is the feeling? Maybe I talk like a therapist.

(14:17 - 14:28)

But you can just say. What do you feel? We also want to start that. I mean, it's just the starting thing, right? I also want to be like them, not only to impress, you know, people.

(14:29 - 15:05)

What do you think is necessary, like, impressing people? I mean, it is necessary. I don't... I mean, that's what we are all, you know, living for. I mean, I... Live.

That's what we all live, I mean, to impress people. Yes. I actually didn't want to do engineering.

I wanted to get into some other course, only, but my parents... You can tell me, which course? I wanted to join civil, like, learn to civil services, and then go to Delhi, prepare for civil services, and then get into UPS. That was my dream. I mean, yeah, my father also wants me to do that.

(15:05 - 15:11)

Still, he wants me to have a secured career. I mean, you know, engineering is the best. Understood.

(15:12 - 15:19)

So he made me get into... Not VIT. Before 10th, before 11th, I had this. So he wanted me to prepare for JEE.

(15:21 - 15:29)

I could have gotten into TIRE 2 and IT, but I chose VIT for this computer science branch. Okay. Okay.

(15:29 - 15:35)

See, here also, I'm only doing this to impress my father. I mean... Understood. Just to impress.

(15:35 - 16:06)

Just impress. To live is to impress, or to impress is to live. I like that.

I'm going to use that. Okay, fine. So, how do you think cultural norms in India impact the way men view their bodies compared to other cultures? Just, you know, like India.

Let's talk about Indian culture. So in Indian culture, we have certain expectations of how a man should look like physically. Okay, so it might be different from the other cultures, like in abroad.

(16:07 - 16:16)

Because, you know, when you watch any movies, or when you see or meet any foreigners, they don't have enough... Beard. Yes, exactly. Yeah, that also comes under... Yes, sir.

(16:16 - 16:23)

Yeah, you tell me. That also comes under physical... You speak. See, this is about you. You can speak. Yes, sir. Yeah.

(16:24 - 16:39)

I remember once I shaved my mustache and then my father scolded me. He scolded not in the way of masculinity, but then he told, that looks bad. See, again, he's expecting me

to look, you know... Manly.

(16:39 - 16:54)

Manly, of course. Because in our culture, like, if you don't have a mustache or beard, they don't approve you of masculinity. Of course, my mother scolds me for having this, that's... See, that's cultural thing, right? Like, mother's like, my mom doesn't like me.

(16:54 - 16:58)

Yes, sir. But now she has changed. If I cut my hair, if I shave, she doesn't like.

(16:59 - 17:26)

You look good with hair. Yeah, that's there. So how do you think, like... Does it impact how you view your body? Like, you know, looking at the people from other cultures, other countries, other socio-cultural environment, when looking at them, does it impact the way you see your body? Anything, like, you talk about this, okay, fine.

(17:26 - 17:40)

Then what about the body, muscles? Anything, like, you can just, you know, think of. Then look at someone, some foreigner, other culture people. Even in India, when I say other cultures, you're from... South India.

(17:40 - 17:56)

South India, like, how does it impact your view? Because we all have different perspectives, right? So I'm from Pondicherry, okay, I'm a Tamil person, okay. So this is how I should be, like, okay. So if you're a North Indian, okay, that's how you should be.

(17:56 - 18:10)

Like, we have this... Even they don't keep me as North Indian. That's how we are. So like, how does it affect the way, the perspective of you seeing your own body? You can just... I also want to, you know, I also want to represent our culture.

(18:11 - 18:25)

See, I just told you, the North Indians, you know, don't have the facial hair. Then I want to grow my hair, I want to represent, you know, see, we look like, we look like this, we, you know, like being like this, of course. Understood.

(18:26 - 18:56)

What about body image? What about the bodies? Just in general, I mean, you understand my question, right? No, can you? So yeah, this understood. What about the overall body image? Like if, for example, if you go to some places, you will see all these people just, you know, not hitting the street. But if you go to some region, they all be beefed up.

(18:56 - 19:03)

Okay, like, how does it influence? So now we are in hostel. So you have different cultures. Okay.

(19:03 - 19:17)

So they all have different notions of being a masculine person, right? So does it, you know, affect you? It is a personal choice, right? It is a personal choice. I don't think that influence. Okay, fine, fine, okay.

(19:18 - 19:30)

So have you ever felt judged or stigmatized based on your physical appearance, particularly concerning your masculinity? Not so far, not so far, but I guess. Not in college. I'm talking about in general.

(19:30 - 19:39)

Like because even relatives, they come home. Yes, yes, yes, yes. That is, you know, just because you look small.

(19:39 - 19:46)

Okay, you know, you're not confident enough or competent enough. They have these perspectives. I have a cousin.

(19:47 - 19:51)

So he looks bigger than me. He looks taller than me. He's like a year younger to me.

(19:51 - 20:00)

And then he looks bigger and taller. So even my, you know, they say you don't, okay, it has nothing to do with masculinity. They're not, you know, doing it with masculinity in this case.

(20:01 - 20:07)

But then they are, you know, comparing us. They're comparing our bodies. And then

they expect me to be like him, you know.

(20:08 - 20:28)

Understood. So like often they judge. Does it often, you know, they also judge you based on your, I mean, judge your intellectual side based on your body? Do you think so? Do you understand? Like when someone looks like a very small kid, but he has very much potential intellectual side.

(20:28 - 20:42)

But when, just by, you know, you know, seeing their body image, they often, you know, get into some conclusion, okay, he's not maybe very much into studies or something. Like, have you ever faced that? I don't think so, sir. I don't think so.

(20:43 - 20:52)

I mean, you know, Preg, Grandmaster, you know how he looks, you know. Still, he's a world champion. Pregnananda, he's a Tamilian chess player.

(20:52 - 20:57)

Oh, yes, yes, yes, yes, yes. Yeah. He's, I don't, I don't think so.

(20:57 - 21:18)

So do you think enough awareness or support available for men who struggle with body image issues? Are you using some? Definitely not. Why is that? Why there is no enough awareness? Like I told, nobody cares. Nobody cares when it comes to men.

(21:19 - 21:36)

See, the attention females are getting, we don't get that, obviously. It's because of our, I guess, it's because of our, you know, historical things that has happened, you know, that made us give more priority, more safety to women. But in 2025, I don't think we need that.

(21:37 - 21:51)

Like, you know, how it was, we know how it was earlier, like before 100, 200 years back, how women were treated. But now they are not being treated same. Still, they are getting that attention, you know, just like if, just like reservation, that was needed earlier.

(21:51 - 21:57)

But now it's not necessary. Still, they are availing that, you know, special powers. Okay.

(21:58 - 22:12)

Yes. Agreed. So how do you think traditional notions of masculinity affect men's mental health, particularly about body image concerns? I told you, I personally have this inferiority complex.

(22:12 - 22:26)

I guess, I guess every 7 out of 10 people have this. What do you do? What do you consider a traditional masculine figure? Of course, a big boy, like a big man with a big body. Like a big man with a big body.

(22:27 - 22:38)

No, it was like very, you know, perfectly uttered. It's a rigid body with a masculine, you know. Yes.

(22:39 - 22:44)

Please, you have to say it. Yeah. Like, okay, I'm showing you three actions, yeah, yeah.

(22:44 - 22:50)

Okay, it's fine. No, no, I understood. Like a big, like a total big, you know, body attire.

(22:53 - 23:00)

Fine. So, okay. How do you think it affects mental health? Yeah.

(23:00 - 23:17)

I use the term inferiority complex for like thrice or fourth. So what happens when you have this? I constantly have that in my mind, you know, whenever I see people, whenever I like think about something that will constantly run in my mind. You know, I need to do this.

(23:17 - 23:24)

I need, I have to do this. Okay, so there is this emphasis of how to do it. Have to do this, yes.

(23:25 - 23:32)

Just to be like that person so that people can accept you. Like, you know, just to impress

people. Impress, accept me more, maybe see me more.

(23:32 - 23:36)

The visibility. Yes. That's what we want.

(23:36 - 23:44)

The visibility, the act of being visible to everyone. Everyone. Without any negative affirmations.

(23:45 - 23:53)

I mean, not negative affirmations, sorry. Negative body image. Okay, when someone looks at you, they don't have to like, dude, this is missing.

(23:53 - 23:58)

This is missing. You have to grow that. I mean, of course, it has nothing to do with me personally.

(23:59 - 24:08)

I am going to be a computer engineer. I should just have my physical health fine. I don't have to have a big body.

(24:08 - 24:16)

See, I have nothing to do with that. I personally have nothing to do with that. But still only to, you know, have that visibility like you told.

(24:16 - 24:23)

I need to do this. I'm not a police to have that physique. I'm not going to be a police.

(24:23 - 24:33)

I'm not going to use it anywhere. That's what I wanted to say. So like we were talking about visibility, right? What about invisibility? Sometimes I'm like, to impress people.

(24:34 - 24:40)

Okay, to impress people, we show things. That is visibility. Like I have pretty muscle.

(24:41 - 24:50)

I'm just going to show, okay. But, you know, if we lack all these said traditional notions of how a body looks like. Okay.

(24:51 - 25:12)

So when we don't have it, when we lack it, we tend to, you know, hide it. So that is invisibility. We are just, so do you do that? Like, do you hide things or your body image? You hide your body image just not to get, you know, get seen by people so that you can avoid all this.

(25:12 - 25:23)

I personally don't, but I've seen relatives posing for pictures. They, you know, put their tummy back, you know, only for that photo. Right after they take that photo, they'll be normal.

(25:24 - 25:49)

See. So what do you believe are the most effective strategies for promoting body positivity and challenging harmful stereotypes about male bodies? Do you think of any strategies, effective strategies for promoting body image, male body image? I don't think having these programs works for this. I don't think.

(25:49 - 25:55)

Just leave all the programs. Just, you know, whatever we do. It has to be within one right.

(25:55 - 26:04)

It has to be within like, like I told you, I've, you know, gone through this. Someone made me feel this. It has to be within them that body shaming is not fine.

(26:04 - 26:09)

No matter if it's a man or a woman. No matter if it's a man or a woman. Body shaming is not fine.

(26:09 - 26:15)

It's not just for women. For men also body shaming is not fine. We also can't take it.

(26:16 - 26:20)

See. It has to be within them. We can't do anything for that.

(26:20 - 26:26)

We can't make them feel that. They have to feel. If you ask me for the strategy.

(26:27 - 26:35)

Yeah, this is a strategy also. Yeah, it's fair enough. For example, like maybe sometimes,

you know, people are.

(26:37 - 26:52)

Okay, like they start, you know, start following all these, you know, Instagram pages about muscles, YouTube videos. They follow all that. They follow all these ideas, you know, macho people.

(26:52 - 26:57)

Okay. So they just start admiring it and they just want to follow it. That's not a strategy.

(26:57 - 27:05)

The strategy is to just, you know, leave yourself out of that place. You don't have to like be obsessed with that, right? Okay. So maybe that is a strategy.

(27:06 - 27:15)

You don't have to immerse yourself in following all these muscle pages. I can do it as long as I don't need it. I don't have to do that.

(27:15 - 27:20)

As long as I don't need it, I don't have to do it. I don't need it. What? Need what? Need that physique.

(27:21 - 27:25)

You know, I'm not a hero. I'm not a hero. I don't have to have that.

(27:25 - 27:30)

I'm not a police. I don't have to use my physique in my, you know, dieting basis. See.

(27:31 - 27:40)

Okay. So are you trying to like maybe build up muscles now? Like are you trying to like... I just want to be fit enough. I don't want to build muscles to have that bulk body.

(27:41 - 27:43)

Just fit. Not like this. Fit enough.

(27:43 - 27:52)

That's it. What do you mean by fit enough? Fit enough. Like what is the body image? I am like right now underweight, right? Sorry? I am underweight.

(27:52 - 27:55)

I cannot say that. I know that. Underweight.

(27:55 - 27:58)

I don't know your weight. For me, you look normal. I am 48.

(27:59 - 28:00)

48. Okay. That's underweight.

(28:01 - 28:04)

I am not 48. I am 43 or something. Sorry.

(28:04 - 28:07)

I am 45. So that's underweight. I just have to be fit.

(28:07 - 28:24)

You know, fit enough. What is fit enough? Please. That, you know, the body height ratio and you have to be this heavy for this, you know, is that is your... How do I say? Defined.

(28:24 - 28:29)

You have to be like that. That's a normal, you know, physics. So I have to attain that.

(28:29 - 28:31)

That's enough. I don't have to. I don't want to build that.

(28:33 - 28:44)

Okay. So from your perspective, what policy changes or societal shifts could help address the gaps in support of men? Okay. Body images.

(28:44 - 28:50)

You have, I think you have already established one thing. How men's face wear blood. Okay.

(28:50 - 29:02)

Why was it always about, you know, women? We don't get, men don't get that attention. Okay. So when it comes to policy making, policy changes or societal shifts, what do you think? Okay.

(29:02 - 29:19)

So I've heard this somewhere. I've heard someone asking a professional some question regarding this, you know, feminism and masculinity. And then he told, I feel when there

is no special rights for women, that is where India has to be.

(29:19 - 29:25)

You know, as long as women have special rights, that's not equality. Hmm. Right.

(29:25 - 29:31)

So when both of them don't have, both of them don't have any rules, that's equality. Okay. I guess.

(29:32 - 29:40)

Okay. When it comes to body image, how are you proposing that? Because, you know, when you see, so I'm just, you know, giving you some examples. Okay.

(29:40 - 29:55)

So when you watch a movie, sorry, advertisements, you see, even for a perfume ad, for any ad, they just, you know, show their body and car. They just, you know, show their six packs. Just for a perfume.

(29:55 - 30:01)

That has to be changed, of course. See, that is also like comes under government only. Okay.

(30:01 - 30:16)

So for what, what to project? Because they all, what is that certification? What is that? Censor. Because they all go through censorship, right? You understand that, like, okay, this is not apt. This is fit for advertising.

(30:16 - 30:21)

No, this is very vulgar. This is controversial. This is, this shouldn't be there.

(30:21 - 30:24)

So they censor everything. Everything has censorship. Okay.

(30:24 - 30:35)

You understand, right? Okay. So when it comes to even, you know, female body, they, you know, for two pieces and they all promote for innerwear, for an underwear. Okay.

(30:35 - 30:46)

So for all the ads, they have this, you know, what do you call a perfect body? A very

muscled ones. Which is not needed. Even for women, even for men, that's how the advertisements goes.

(30:47 - 30:57)

If you go for Junkie or any other popular brands, they all have this, you know, conventional, not conventional, sorry. They have this like traditional. Oh, this is a home.

(30:58 - 31:14)

So to wear this underwear, to wear this innerwear, you have to be like properly shaped. Okay. What is the, I don't think like the people, I don't think people who possess the whatever that was screened, they are not the only consumers.

(31:14 - 31:21)

Okay. If they see, I don't look like the one that was on the ad, but I still purchase Junkie. Okay.

(31:21 - 31:24)

I still purchase that cloth. You understand. I still purchase that perfume.

(31:24 - 31:27)

All the stereotypes. Yeah. So stereotypes, right.

(31:28 - 31:42)

So maybe what do you think about that policy? Like, do you like recommend something? Maybe change, instead of just having all this. Yeah. This is a good example.

(31:43 - 31:55)

Yeah. Instead of having all these, you know, like better bodies with popular figures, I mean actors and actresses, the inclusivity should be there, right? All body forms. Okay.

(31:55 - 32:20)

Even if an oversized men, women can be presented in that ad as well. So do you think this could, you can just, you know, say, do you have anything? So yeah, what is the, what could be the policy? I mean, the change should start from the individual or their perception on themselves. That has to be within them.

(32:20 - 32:34)

So maybe the government can have, you know, policies to create awareness, to make

them understand that it's to make the people understand that it's fine to have, you know. Different body shapes. Yeah.

(32:34 - 32:41)

Yes. It's fine to have different bodies. Thank you.

(32:41 - 32:47)

So you, what's your age? I'm 18. 18. Cisgender male, you understand all this concept, right? Okay.

(32:47 - 32:52)

Male. What is your sexual orientation? I'm a male. Straight, heterosexual.
